# Clinical utility of PDX cohorts to reveal biomarkers of intrinsic resistance and clonal architecture changes underlying acquired resistance to cetuximab in HNSCC

**DOI:** 10.1038/s41392-022-00908-0

**Published:** 2022-03-08

**Authors:** Yanli Yao, Yujue Wang, Lan Chen, Zhen Tian, Guizhu Yang, Rui Wang, Chong Wang, Qi Wu, Yaping Wu, Jiamin Gao, Xindan Kang, Shengzhong Duan, Zhiyuan Zhang, Shuyang Sun

**Affiliations:** 1 Department of Oral and Maxillofacial-Head Neck Oncology, Shanghai Ninth People’s Hospital, Shanghai Jiao Tong University School of Medicine, Shanghai, China; 2 College of Stomatology, Shanghai Jiao Tong University; National Center for Stomatology; National Clinical Research Center for Oral Diseases; Shanghai Key Laboratory of Stomatology, Shanghai, China; 3 Department of Oral Pathology, Shanghai Ninth People’s Hospital, Shanghai Jiao Tong University School of Medicine, Shanghai, China; 4 Laboratory of Oral Microbiota and Systemic Diseases, Shanghai Ninth People’s Hospital, Shanghai Jiao Tong University School of Medicine, Shanghai, China

**Keywords:** Head and neck cancer, Tumour biomarkers

## Abstract

Cetuximab is a widely used drug for treating head and neck squamous cell carcinomas (HNSCCs); however, it provides restricted clinical benefits, and its response duration is limited by drug resistance. Here, we conducted randomized “Phase II-like clinical trials” of 49 HNSCC PDX models and reveal multiple informative biomarkers for intrinsic resistance to cetuximab (e.g., amplification of *ANKH*, up-regulation of PARP3). After validating these intrinsic resistance biomarkers in another HNSCC PDX cohort (61 PDX models), we generated acquired cetuximab resistance PDX models and analyzed them to uncover resistance mechanisms. Whole exome sequencing and transcriptome sequencing revealed diverse patterns of clonal selection in acquired resistant PDXs, including the emergence of subclones with strongly activated RAS/MAPK. Extending these insights, we show that a combination of a RAC1/RAC3 dual-target inhibitor and cetuximab could overcome acquired cetuximab resistance in vitro and in vivo. Beyond revealing intrinsic resistance biomarkers, our PDX-based study shows how clonal architecture changes underlying acquired resistance can be targeted to expand the therapeutic utility of this important drug to more HNSCC patients.

## Introduction

Head and neck squamous cell carcinomas (HNSCCs) develop from the mucosal epithelium in the oral cavity, pharynx, and larynx and are the most common malignancies that arise in the head and neck. Epidermal growth factor receptor (EGFR) is amplified and overexpressed in most (≥80%) HNSCC tumors, and is associated with more aggressive disease and poorer prognosis.^1^ Cetuximab is a chimeric EGFR IgG_1_ monoclonal antibody (mAb) approved in combination with radiation therapy for the treatment of locally advanced HNSCCs, and in combination with platinum-based chemotherapy for treatment of recurrent and/or metastatic HNSCCs.^2,3^ Despite the improvement in clinical outcomes for HNSCCs as the result of cetuximab combination therapies, intrinsic or acquired resistance increases tumor recurrence rates and limits clinical efficacy.^4^ Notably, the treatment efficacy of cetuximab is low, with an objective response rate of 13% in the monotherapy setting^2^ and 36% in combination with chemotherapy.^5^ Time-to-treatment failure in patients treated with the EXTREME regimen ranges only around 5 months despite cetuximab maintenance.^4,6^ Importantly, strategies for blocking EGFR achieve major tumor regressions in ~10–20% of advanced cancer patients.^7^ However, the majority of patients who initially respond to cetuximab eventually manifest acquired resistance to treatment.^8^

Drug resistance is a huge problem in cancer therapy and a barrier to improving survival outcomes for many patients.^9,10^ Numerous studies have revealed that cancer drug resistance is determined not by singular, static, mutually exclusive alterations, but rather by a multifactorial, heterogeneous, and “evolutionary” landscape, and noted that studies of cancer drug resistance are prone to undersampling at a specific time or location.^10–12^ In addition, the burgeoning norm of using multiple drug treatments has also added substantial complexity to reductionist approaches to research the development of patient resistance to a given monotherapy.^9,13^ Thus, innovation in research methods that can account for the complexity of drug resistance would be welcomed and could likely facilitate future therapeutic insights.

Patient-derived xenograft (PDX) models can be established within highly immunocompromised mice; these models have been shown to maintain the morphological and molecular characteristics of the original heterogeneous patient tumors^14,15^ and faithfully predict patient drug responses.^15,16^ In 2011, Bertotti et al. tested cetuximab against a set of colorectal cancer (CRC) PDX models and reported strong response concordance between the PDX models and the corresponding CRC patients in the clinic.^17^ In 2015, Guo et al. performed in vivo compound screens using a 1 × 1 × 1 (one animal per model per treatment) PDX clinical trial (PCT) to assess population-level responses of 62 treatments across six cancer indications, and proposed that this experimental paradigm can informatively predict therapeutic responses.^18^ Later studies adopted PCT approaches for successful “phase II-like preclinical trial studies”, for example, B-cell acute lymphoblastic lymphoma (B-ALL) PCT was used for preclinical assessment of MDM2 inhibitor,^19^ as well as the pediatric tumor PCT for 67 agents across 83 xenograft models.^20^

Extensive evidences have emphasized that the experimental paradigm of PCT is an effective approach for drug resistance research, which can both improve preclinical evaluation of treatment modalities and enhance the ability to informatively predict therapeutic responses.^19,21^ More sophisticated investigations are also possible: PDXs can be treated up to the point where they progress, which allows for the experimental development of acquired resistance.^14^ Samples can then be taken by euthanizing sentinel animals at multiple time points to establish biomarkers of response. It is also possible to carry out clonal evolution research, which can support translational studies of the mechanisms of acquired resistance in specific patient tumors and can help determine optimal personalized therapies to combat acquired resistance.^14,15,22^

Here, after establishing a biobank of HNSCC PDX models, we first investigated potential biomarkers for intrinsic resistance to cetuximab in HNSCCs based on two independent PCTs. With paired samples collected from pre-treatment, on-treatment, and post-treatment, we mapped the cetuximab-induced evolutionary trajectories and found diverse patterns of clonal selection in the acquired resistance PDXs. We demonstrated that a combination therapy comprising the RAC1 and RAC3 dual-target inhibitor EHOP-016 and cetuximab confers strong and synergistic in vitro and in vivo efficacy against HNSCCs. Thus, beyond its identification of informative biomarkers for intrinsic cetuximab resistance, our study illustrates how dynamic changes in transcriptomes and clonal architecture underlie acquired resistance to cetuximab in HNSCCs.

## Results

### Anti-tumor activity profile of cetuximab in a population-level PDX clinical trial

We undertook efforts aiming to build a biobank of surgical materials from HNSCC patients, which were stored under viable conditions and serially propagated in mouse recipients. In total, a large-scale panel of HNSCC PDXs were established and were amenable to serial passaging. Whole exome sequencing (WES) and RNA sequencing (RNA-seq) were performed on the representative tumor grafts, the parental tumors, and matched normal tissues. The results showed that HNSCC PDXs exhibit a high degree of genomic stability and gene expression pattern similarity in comparison with their parental tumors (Supplementary Results, Fig. S1).

This large-scale panel of PDX models enables population-based “Phase II like” studies, using several animals per model per treatment group. We randomly selected 49 HNSCC PDXs and conducted a PCT to interrogate the interpatient response heterogeneity, aiming to evaluate the efficacy of cetuximab monotherapy for treating HNSCCs in the population. A summary of the detailed clinical information for the randomly selected PDX models from 49 patients that were enrolled in our PCT is provided in Supplementary Table 1.

**Fig. 1:**
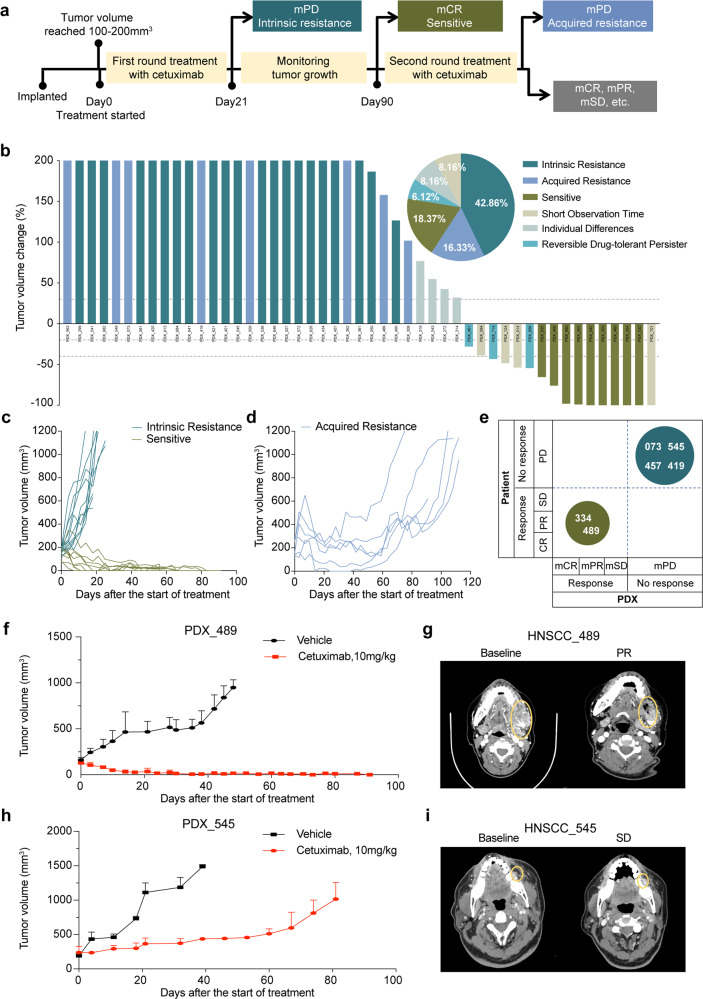
Anti-tumor activity profile of cetuximab in a randomized population-level PDX clinical trial in HNSCCs. **a** Workflow for the randomized PCT of cetuximab. Drug response phenotypes were assigned as “sensitive”, “acquired resistance”, or “intrinsic resistance” based upon the tumor volume change. Twenty-one days after the first-round treatment initiation, mPD cases were classified into the intrinsic resistance group. mCR cases that did not relapse within 90 days after the first-round treatment initiation were classified into the sensitive group. Relapsed cases were classified into the acquired resistance group if they had an mPD phenotype in the third week after the second drug treatment initiation. **b** Waterfall plot of the PDX average response to cetuximab, defined as tumor volume change compared with tumor volume at baseline (Day0); each bar represents an individual PDX (*n* = 49). Dotted lines indicate the cutoff values for arbitrarily defined categories of therapy response, as follows: intrinsic resistance (dark blue), acquired resistance (blue), sensitive (dark brown), reversible drug-tolerant persister (light green); individual differences (light aquamarine), short observation time (light grey). The *x* axis represents each case, and the *y* axis represents the tumor volume change. The maximum threshold for tumor volume change was set at 200%. The minimum threshold for tumor volume change was set at −100%. The pie chart at the top right shows the distribution (as proportions) of the different cetuximab-response groups (*n* = 49). **c**, **d** Representative tumor volume growth curve in 49 cases of PDXs treated with cetuximab. Tumor volume growth curve for the sensitive and intrinsic resistance groups (**c**) and the acquired drug resistance group (**d**). **e** Representation of PDXs versus patient responses for the 6 cases in our PCT. The size of the circles represents the number of subjects in each response category. **f** Tumor growth of PDX_489 treated with cetuximab and PBS as single agents. **g** CT scans showing the anti-EGFR monoclonal antibody response of patient HNSCC_489. **h** Tumor growth of PDX_545 treated with cetuximab and PBS as single agents. **i** CT scans showing the anti-EGFR monoclonal antibody response of patient HNSCC_545. Yellow circles indicate the tumor localization

A PCT on the cohort of 49 HNSCC PDXs was conducted (“*n* = 3 format”: three mice per arm, two arms: vehicle and cetuximab treatment) to assess the cetuximab efficacy. In this experimental setup, we denominate a “case” as the average performance (see below) of 3 PDX models from one patient. The first evaluation was scheduled 3 weeks after treatment initiation. We used tumor volume change values to classify each case into one of three groups with the following pre-determined criteria: (i) Progressive disease (mPD) cases will be classified as the intrinsic resistance group. (ii) Complete response (mCR) cases will be monitored for up to 90 days; if the tumor does not relapse, the cases will be classified as the sensitive group. (iii) If there is any relapse for the following response categories—mCR, as well as suboptimal stabilization (mSD) and partial response (mPR)—during the initial 90 days of treatment, the tumor will be collected as a recurrence sample and passaged (mice were euthanized due to the humane endpoint), followed by the second round of cetuximab treatment. The mPD cases identified in this second round will then be classified as the acquired resistance group. Cases where recurrence occurred afther the first-round treatment but mPR or mCR occurred in the second round of treatment, indicated that cancer cells entered a reversible drug-tolerant persister (DTP) state to evade death from targeted agents^23^ (Fig. 1a).

Tumor volume changes after cetuximab treatment (compared with baseline) are summarized in Fig. 1b–d, Supplementary Fig. 2, and Supplementary Table 2. There were 21 cases in the intrinsic resistance group (42.86%), 9 cases in the sensitive group (18.37%), and 8 cases in the acquired resistance group (16.33%); there were also 4 cases with huge individual differences (8.16%), 4 cases (8.16%) were stopped midway due to severe side effects in the mice, and 3 cases (6.12%) were reversible drug-tolerant persisters (Fig. 1b). Collectively, these response rates for cetuximab are consistent with those obtained in clinical trials: the reported objective response rate is only 13% when cetuximab is used as a single agent.^2^

These PCT findings underscore the clear need for biomarkers that are predictive of response to cetuximab to help maximize the likelihood of a therapeutic response. Notably, we also observed the PDX models in our PCT recapitulated patient responses (Fig. 1e–i). For example, PDX_073—derived from a progression gingiva squamous cell carcinoma sample, in a 56-year-old male. He received cetuximab–docetaxel–cisplatin for 21 days—exhibited a short stable disease until tumor recurrence. PDX_073 mimics the acquired drug resistance observed in the patient: with mSD (−10.13%) under cetuximab treatment in the first treatment and then relapse within 38 days, finally reaching mPD (564.36%) (Fig. 1e). The parental patients of PDX_489 and PDX_545 received anti-EGFR therapy, HNSCC_489 exhibited clinical benefit (−52.12%) and HNSCC_545 had a modest tumor response (−5.08%) and a brief progression-free survival (~1 month). The corresponding PDXs mirrored patient tumor sensitivity to anti-EGFR treatment, and the tumor response of PDX_489 and PDX_545 were mCR (−100.00%) and mPD (352.76%) (Fig. 1f–i), respectively. These results emphasize that clinical outcomes can be recapitulated in our PCT.

### Genetic alterations and transcriptome characteristics associated with cetuximab response and biomarker analysis in HNSCC PCT

Our PDX models can recapitulate major complexities of patients’ malignancies, including their responses to cetuximab. To identify genetic alterations and transcriptome characteristics associated with the cetuximab response that could be predictive biomarkers, we used both RNA-seq and WES to analyze the PDX models of the present study. We selected matched samples for sequencing based on their cetuximab responses, including nine cases in the intrinsic drug-resistant group, seven cases in the sensitive group, and eight cases in the acquired drug resistance group. All of the corresponding patients’ tumor and adjacent tissues for these 24 cases were included as controls.

An advantage of working with PDX models is the capacity to characterize the molecular basis of a cetuximab response during treatment. Such investigations are not yet routinely performed in patients, owing to clinical challenges in deciding which tumor site to re-biopsy, as well as ethical issues such as the invasiveness of the biopsy procedure. With PDXs, however, multiple biological replicates can be implanted, allowing for subsets to be studied at various time points of the treatment regimen. We thus included pre-treatment and post-treatment samples for the intrinsic resistance and acquired resistance PDXs, as well as the relapsed samples in the acquired resistance group for both WES and RNA-seq.

**Fig. 2:**
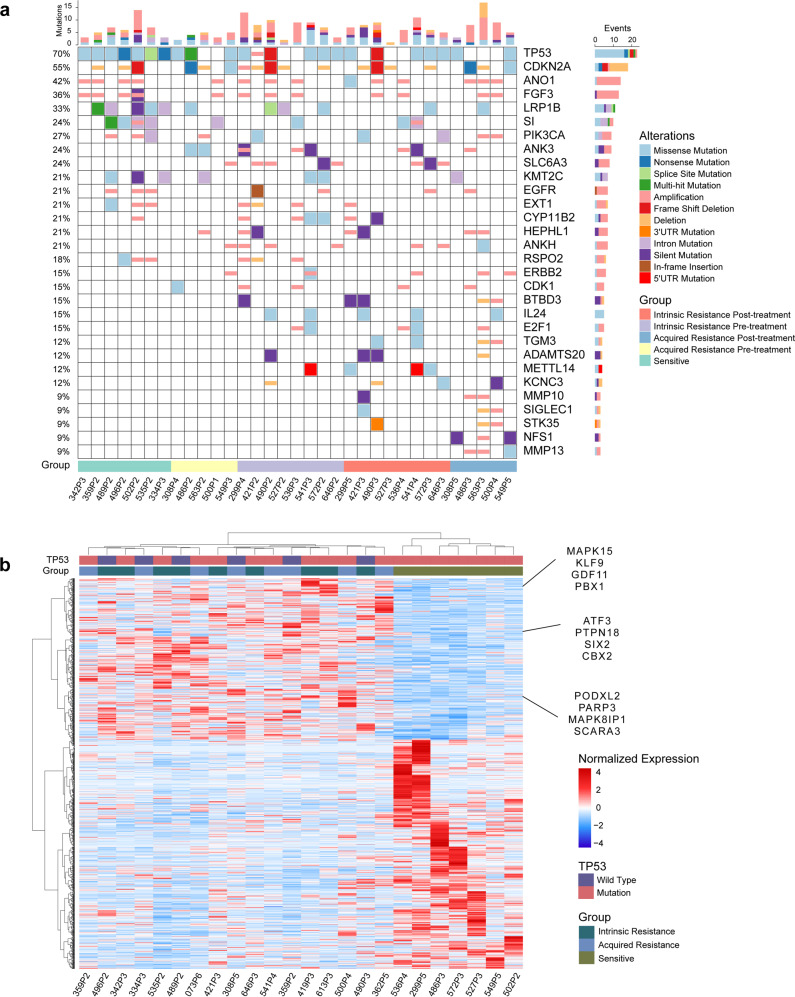
Genetic and transcriptomic landscape of HNSCC PCT. **a** Genetic matrix depicting the genomic landscape of the cetuximab PCT cohort. Copy number alterations and mutational events in select genes of interest are shown. Parameters (shown at the right) include alterations and groups. CNV, copy number variation; SNV, single-nucleotide variant. The *x* axis represents each sample ID and the *y* axis represents the frequency of CNVs and SNVs in the genes. The bar graph indicates the number of gene alterations in each sample. **b** Unsupervised hierarchical clustering heatmap showing the relative gene expression levels of the 2500 selected variable genes from an RNA-seq analysis of resistant and sensitive PDXs. Twelve genes of interest are labeled on the right side of the heatmap

For the WES data, analyses of single-nucleotide variants (SNVs) and of copy number variants (CNVs) indicated that increased drug resistance is often accompanied by an increasing trend for mutations in candidate cancer genes in the Network of Cancer Genes^24^, such as *ERBB2*, *IL24*, *E2F1*, *CDK1, MMP10, MMP13,* etc. It was highly notable that none of these genes had SNVs or CNVs in the sensitive group; however, the variation rates for these genes ranged from 15.38 to 30.77% in the intrinsic and/or acquired resistance group (Fig. 2a). Previous studies have reported *ERBB2* amplification as a potential mechanism that could underly cetuximab resistance.^25^ In our PCT study, 19.23% of the intrinsic and acquired resistant cases harbored amplification of *ERBB2*, whereas no SNVs or CNVs were detected in the sensitive group.

In the intrinsic and acquired resistant group, *IL24* showed a 19.23% mutation rate, while the sensitive group had no mutations at this locus. We also found two potential new cetuximab resistance predictive markers: *ANK3* and *ANKH*. The mutation rates of these two genes in the TCGA HNSCC database were only 3.50% and 1.50%, and the frequency of copy number variations was less than 4.00%. In contrast, our PCT study showed that 30.77% and 26.92% of the intrinsic and acquired resistant PDX models had alterations in *ANK3* and *ANKH*, respectively. No *ANK3* or *ANKH* mutations were detected in the sensitive group. We also found that genes with functions in RAS signaling were over-represented in the intrinsic and acquired resistant groups (Supplementary Fig. 4b), suggesting that RAS signaling somehow contributes to the emergence of cetuximab resistance. In addition, in our PCT, we comprehensively analyzed previously reported SNVs or CNVs associated with cetuximab resistance (mainly in CRCs) and failed to find a clear link between them, which indicates that there could be distinct biomarkers and drug resistance mechanisms in HNSCCs that differ from those in CRCs (Supplementary Fig. 3a).

We found that the mutation rate of *TP53* in the sensitive group was 100.00% but was only 62.50% in the intrinsic resistance group, indicating that cases with *TP53* mutations might be more sensitive to cetuximab. This notion was further supported in our comparisons of *TP53* mutations before and after administration in the acquired resistance group (Supplementary Fig. 4a). It was also interesting to note that the specific site of the *TP53* mutation could influence the cetuximab response. In other words, the R150W and R174X *TP53* mutations in the sensitive group were not detected in the intrinsic resistance group. For the acquired resistance models, we detected R150W and R174X mutations before administration, but these were not present in these models when we sampled after administration. These results suggest that *TP53* mutations might informatively predict sensitivity to cetuximab (Supplementary Fig. 4a).

We also conducted transcriptome sequencing to help identify intrinsic molecular mechanisms of HNSCCs that could contribute to cetuximab resistance. The sensitive PDXs and the resistant PDXs exhibited significantly different gene expression distributions, and it was notable that genes abnormally overexpressed in multiple cancer types, such as *PBX1*, *ATF3*, *PARP3*, and *PTPN18* were among the top-ranking differentially expressed genes in the intrinsic resistance group (Fig. 2b and Supplementary Fig. 3b).

### Cross-validation of predictive biomarkers for cetuximab resistance in an independent PCT

The ability of PDXs to closely mimic human cancer from which they were derived makes them a valuable tool in biomarker discovery and validation. To evaluate whether the identified biomarkers were associated with the response to cetuximab, we examined tumor graft responses to cetuximab therapy for 61 PDXs and implemented another independent PDX clinical trial. A summary of the detailed clinical information for the 61 randomly selected PDXs from 61 patients who were enrolled in our PCT is provided in Supplementary Table 3.

**Fig. 3:**
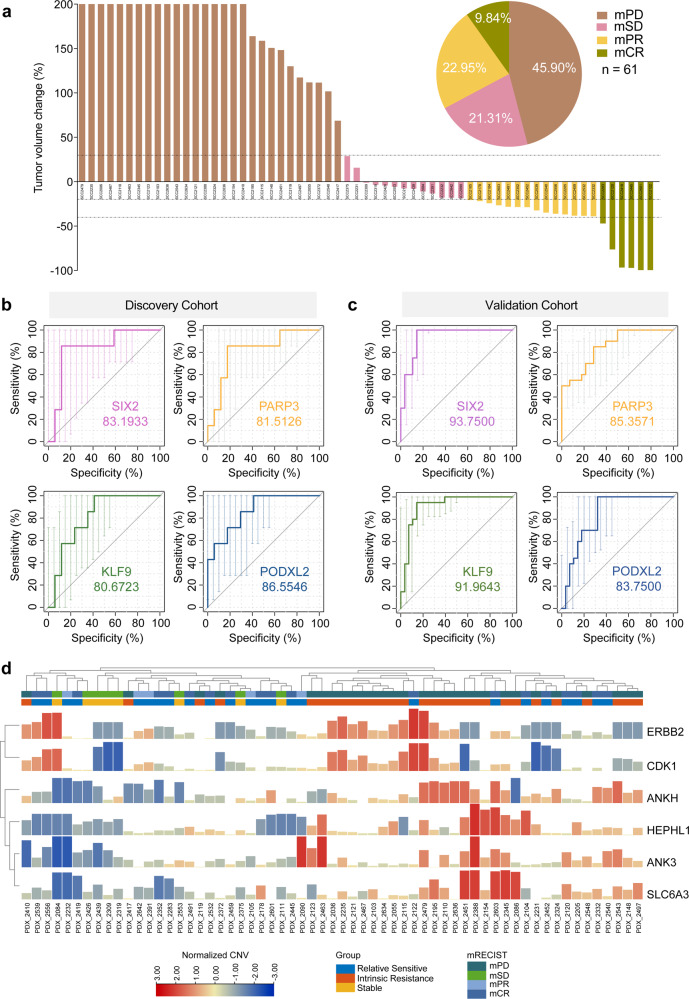
Identification of predictive biomarkers for cetuximab resistance in an independent PDX cohort. **a** Waterfall plot of cetuximab response in the validation PDX cohort, defined as the tumor volume change compared with tumor volume at baseline after 3 weeks from treatment started (*n* = 61). Dotted lines indicate the cutoff values for arbitrarily defined categories of therapy response: mPD, mSD, mPR, and mCR are shaded in dark orange, light purple, light orange, and dark brown, respectively. The pie chart shows the distribution (as proportions) of the different cetuximab-response groups (*n* = 61). **b** Receiver operating characteristic (ROC) curves of discovery cohort measured by RNA sequencing of SIX2, PARP3, KLF9, and PODXL2 in distinguishing the sensitive group and the intrinsic resistance group. **c** The ROC curves of the validation cohort measured by real-time qPCR of SIX2, PARP3, KLF9, and PODXL2 in distinguishing the relatively sensitive group and the resistance group. **d** CNV profiles of intrinsic resistance and relatively sensitive PDX samples in the validation PCT. Each sample ID is denoted by the PDX model ID

The volume of each tumor graft was evaluated twice a week for 21 days after initiation of treatment, and tumors were categorized using the same method described above for the first cohort. Among tumor grafts, 28 cases (45.90%) were disease progression (mPD), 13 cases (21.31%) with suboptimal stabilization (mSD), 14 cases (22.95%) with partial response (mPR) and 6 cases (9.84%) with complete response (mCR). The mPD PDXs were defined as the intrinsic resistance group, and the mCR and mPR PDXs were defined as the relatively sensitive group (Fig. 3a and Supplementary Table 4).

Working with this validation cohort, we conducted Sanger sequencing of amplified DNA, copy number variation detection, and qPCR analysis of the expression levels of a set of 30 potential biomarkers that we initially identified in the discovery cohort. Receiver operating curves (ROCs) comparing cohort subgroups provided the AUC for cetuximab resistance biomarker positivity. High expression of *SIX2*, *KLF9*, *PARP3*, and *PODXL2* were thusly verified as predictive biomarkers in both the discovery and validation cohorts: these biomarkers each discriminated between relatively sensitive cases and intrinsic resistance cases with high accuracy (AUC > 0.80) (Fig. 3b, c and Supplementary Fig. 5a–c). The copy number variation test identified amplification events for the *ERBB2*, *CDK1*, *ANKH*, *HEPHL1*, and *SLC6A* loci as resistance biomarkers in the validation cohort (Fig. 3d). We also examined the potential cetuximab-sensitive mutation sites in *TP53* (encoding R150W and R174X mutations): 1 mCR case (16.67%) harbored an R150W mutation (Supplementary Fig. 5d). These results support the utility of PCTs and related molecular investigates for identifying and validating biomarkers that can informatively predict intrinsic resistance to cetuximab.

### Clonal evolutionary dynamics in acquired resistance to cetuximab based on HNSCC PCT

Previous drug resistance studies have provided evidence that both intratumoral heterogeneity and the evolutionary pressure of therapy-induced clonal selection can promote acquired drug resistance.^11,26,27^ Clonal architecture can be conserved between patient tumors and case-matched PDXs,^22^ and thus, we used serially collected PDX samples to help elucidate clonal architecture dynamics during the development of acquired resistance to cetuximab treatment.

**Fig. 4:**
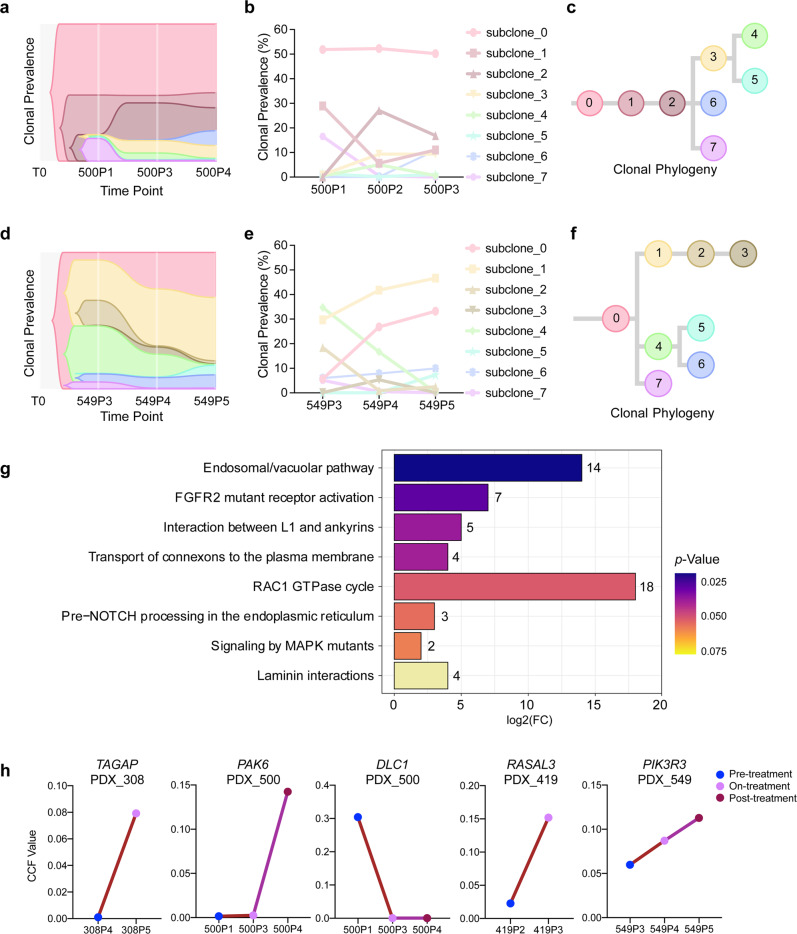
Clonal structure and phylogenetic reconstruction of PDXs in the acquired resistance group. **a**, **d** Clonal evolution in response to cetuximab treatment inferred from WES sequencing. Plots of clonal lineages and frequency changes over time and in response to cetuximab treatment for two cases (PDX_500 and PDX_549). **b**, **e** The cellular prevalence of mutation clusters across PDX samples identified by PyClone analysis. The founding clone is indicated in light red as subclone_0, with subsequent subclones shown in distinct colors. The line graphs show the clonal prevalence of each subclone in multiple samples from the same case (corresponding to the area of the phylogenetic trees). **c**, **f** Phylogenetic trees constructed based on the mutation clusters for PDX_500 (**a**) and PDX_549 (**d**) in the acquired resistance group. **g** Analysis of significantly differentially enriched pathways in newly generated subclones in the acquired resistance group. **h** The line graphs illustrate changes in the CCF value of gene mutations (*TAGAP, PAK6, DLC1, RASAL3, PIK3R3*) through cetuximab treatment in acquired resistance group

Matched pre-treatment and post-treatment samples were available from six cases who experienced acquired resistance to cetuximab, of these, two cases included pre-treatment, on-treatment (recurrence), and post-treatment (acquired resistance) samples. To explore the cetuximab resistance mechanism, we used PyClone to integrate the WES data from these paired samples and used CITUP to infer the clonal evolution across different time points.^28^ We established the evolutionary classification of each mutation to distinguish events that were enriched or reduced in subclones that are dominant in the post-progression tumor (as compared with its pre-treatment counterpart). We identified multiple subclones (range 5–7) in the 6 cases and identified dynamic changes in clonal frequencies in response to cetuximab (Fig. 4a–f and Supplementary Fig. 6a–d). All acquired resistant samples showed multiple subclones, and several subclones were usually found in pre-treatment tumors, with a few subclones introduced into a subset of the progeny, termed branched events, suggesting that acquired resistance occurs via polyclonal seeding, not a single cell, mainly as classical models of branched tumor evolution.^29^

In our branching model, we analyzed the subclone dynamics and identified three distinct responses of the subclones to cetuximab: (1) newly generated subclones; (2) eliminated subclones; and (3) persistent subclones. Mutations in the newly generated subclones were significantly associated with acquired resistance to cetuximab. For example, in PDX_073, a minor subclone (subclone 5) with <1% clonal frequency in the initial timepoint (pre-treatment) expanded to 26% at the final timepoint (post-treatment) (Supplementary Fig. 6a). The eliminated subclones disappeared completely after treatment with cetuximab, suggesting that these subclones were sensitive to cetuximab. In PDX_486, a subclone (subclone 3) with 23% clonal frequency at the initial timepoint was reduced to <1% in response to cetuximab therapy (Supplementary Fig. 6b). Persistent subclones were detected both pre-treatment and post-treatment, but had either decreased or increased frequencies in response to cetuximab. In PDX_563, for example, three persistent subclones were detected. The clonal frequency of subclone 2 decreased from 26 to 23% in response to cetuximab, while subclone 4 increased from 11 to 17%, and subclone 3 maintained its ~27% clonal frequency (Supplementary Fig. 6d).

We analyzed all of the newly generated subclones in the recurrence and acquired resistance samples among six cases and found a total of 410 unique mutations, among which 2 mutations (*SREK1* and *ATG2A*) were shared by three cases and 17 mutations (*NOTCH3*, *NOTUM*, *NFS1*, etc.) were shared by 2 cases (Supplementary Fig. 7a). We also analyzed the eliminated subclones among the six cases: there were 454 lost mutations, of which 1 mutation (*ZNF717*) was lost from three cases (PDX_486, PDX_563, and PDX_549), and 14 mutations (*OR10A2, TEX14, MAP4K1*, etc.) were lost from two cases (Supplementary Fig. 7b). Our data indicated the low frequencies of common mutation alterations, which highlights the broad heterogeneity of HNSCCs.

It is plausible that the newly generated or eliminated subclones resulting from cetuximab drug pressure selection could have contributed to acquired resistance;^11^ as a result, we performed pathway enrichment analysis with the Reactome Database for the mutated genes among the newly generated and eliminated subclones. The 864 mutated genes (410 in newly generated subclones; 454 in eliminated subclones) were enriched for annotations related to several cancer-related pathways, including “FGFR2 mutant receptor activation”, “RAC1 GTPase cycle”, “Signaling by MAPK mutants”, etc. (Fig. 4g).

Notably, the RAC1 GTPase cycle pathway is enriched. RAC1 (Ras-related C3 botulinum toxin substrate 1) has been directly implicated in the morphogenic and mitogenic responses of tumors to transformation in response to the presence of the oncogenic protein Ras.^30^ In PDX_308, *TAGAP*, the gene encoding the GTPase-activating protein,^31^ was mutated after treatment with cetuximab in newly generated subclone 7 (Fig. 4h). In PDX_500, *PAK6* (RAC1 activated kinase 6)^32^ was of the wild-type allele in both the pre-administration and recurrence samples, whereas a mutation occurred in newly generated subclone 6 (in the post-administration samples) (Fig. 4h). Furthermore, we detected a change in the tumor suppressor gene *DLC1*—which has been shown to inactivate small Rho GTPase proteins^33^—in the eliminated subclone 7: it changed from a mutant allele to the wild-type during the process of acquired drug resistance, resulting in the restoration of its activity, thereby promoting the elimination of subclone 7 (Fig. 4h).

### Repeated evolutionary trajectories of acquired resistance of cetuximab based on HNSCC PCT

A phylogenetic tree comprising each acquired resistance case constructed using PyClone and CITUP was extremely heterogeneous, and thus, it was difficult to ascertain repeated evolutionary trajectories occurring during acquired resistance development. We, therefore, applied transfer learning with REVOLVER^34^ to denoise and structurally correlate trees across the eight acquired resistance cases to determine whether any of the pre-administration PDXs or post-administration PDXs shared common evolutionary trajectories that reflected a constrained clonal mutation order. This analysis sought to define potential bottlenecks that could be targeted for helping to overcome acquired resistance to cetuximab.

**Fig. 5:**
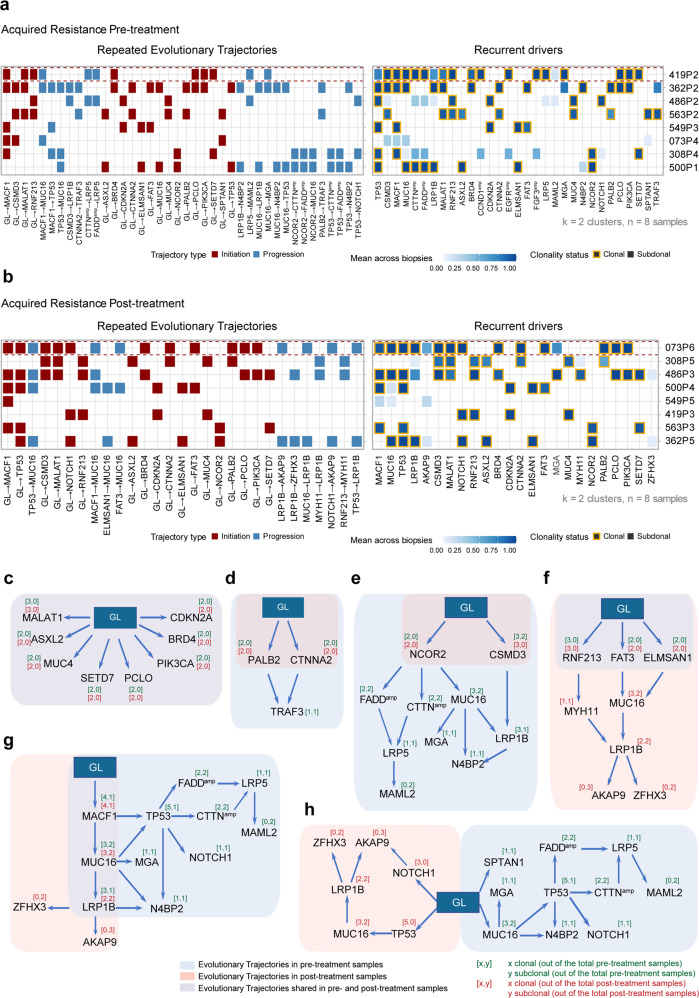
Repeated evolutionary trajectories of acquired cetuximab resistance in HNSCCs. **a**, **b** REVOLVER analysis of pre-treatment samples (**a**) and post-treatment samples (**b**) from acquired resistance group. The left heatmap shows the most common repeated evolutionary trajectories, and the right heatmap shows the average proportion of samples bearing the alteration for the most recurrent putative driver genes (on the basis of presence/absence). **c** Repeated evolutionary trajectories unchanged before and after cetuximab administration. **d**, **e** Repeated evolutionary trajectories retained only the early transition after cetuximab administration. **f** Repeated evolutionary trajectories with early transitions extended after cetuximab administration. **g** Cetuximab retained the backbone of the repeated evolutionary trajectories of pre-administration samples but had a different extension on *LRP1B* mutation. **h** Repeated evolutionary trajectories that disappeared and were reborn after cetuximab treatment. GL stands for germline. Arrows indicate transitions. The number of times an alteration was clonal or subclonal in the cetuximab acquired resistant samples

Integrating both clonal SNV and CNV driver events, REVOLVER produced several repeated evolutionary transitions for the eight pre-administration samples and eight post-administration samples (Fig. 5a, b and Supplementary Fig. 8a, b). Notably, the evolution trajectory network of the pre-administration samples was obviously more complex than the trajectory of the post-administration samples, which likely reflects the coexistence of both sensitive subclones and resistant subclones in these samples (Supplementary Fig. 8a, b). There were eight common repeated early clonal transitions in both the pre- and post-treatment samples that were consistently found as early clonal events, which were likely involved in acquired resistance initiation (germline (GL) → *CDKN2A*; GL → *PIK3CA*; GL → *BRD4*; GL → *SETD7*; GL → *MUC4*; GL → *PCLO*; GL → *MALAT1*; GL → *ASXL2*) (Fig. 5c).

The evolutionary trajectories that disappeared in the post-administration samples under drug pressure selection were associated with cetuximab sensitivity (Fig. 5d, e, g). *NCOR2* is a node involved in five evolutionary trajectories for the pre-administration samples and with subsequent *MUC16* mutation, *FADD* amplification or *CTTN* amplification, etc. However, only the early clonal transition of GL → *NCOR2* was detected for the post-administration samples (Fig. 5e). Similarly, the downstream evolutionary trajectory transited by *NCOR2* disappeared after cetuximab administration, leaving the early clonal transition of GL → *NCOR2* (Fig. 5e). A *TRAF3* inactivating mutation was previously reported to be associated with a better prognosis in HNSCCs.^35^ We also found that two evolutionary trajectories leading to *TRAF3* mutation disappeared after cetuximab administration (GL → *CTNNA2* → *TRAF3*, GL → *PALB2* → *TRAF3*) (Fig. 5d).

The newborn evolutionary trajectories in post-administration samples (acquired resistance) could be associated with cetuximab resistance (Fig. 5f–h). The repeated evolutionary trajectory GL → *NOTCH1* → *AKAP9* was acquired after treatment, with the *NOTCH1* mutation changing from a late event in the pre-treatment samples to an early event in the post-treatment samples (Fig. 5g, h), indicating that the *NOTCH1* mutation might drive the development of acquired drug resistance. Previous research has reported that the *NOTCH1* mutation is a predictive factor for anti-CD20 monoclonal antibody resistance in chronic lymphocytic leukemia in a randomized chemo-immune therapy phase III trial (COMPLEMENT 1).^36^

Interestingly, we found that five nascent evolutionary trajectories (GL → *RNF231* → *MYH11* → *LRP1B* → *AKAP9/ZFHX3*; GL → *FAT3* → *MUC16* → *LRP1B* → *AKAP9/ZFHX3*; GL → *MACF1* → *MUC16* → *LRP1B* → *AKAP9/ZFHX3*; GL → *ELMSAN1* → *MUC16* → *LRP1B* → *AKAP9/ZFHX3*; GL → *TP53* → *LRP1B* → *AKAP9/ZFHX3*) all ended up pointing to the same late events (*LPR1B* mutation accompanied by *AKAP9* or *ZFHX3* mutation) (Fig. 5f–h and Supplementary Fig. 8b) in post-treatment samples. The zinc-finger homeobox 3 protein ZFHX3 has been reported to function as a tumor suppressor in several cancers, and *ZFHX3* mutation defects are associated with poor outcomes.^37^ ZFHX3 is a bona fide repressor of MYC transcription in prostate cancer, and the loss of *ZFHX3* can dramatically increase MYC expression, which is a critical effector of RAS-driven cancer.^37^ RAS and MYC are canonical examples of cooperating oncogenes: these proteins coregulate a broad set of gene programs that are essential to growth, differentiation, and proliferation.^38^ In addition to the widely appreciated role of activated RAS driving cancer cell signaling through binding to effector proteins, constitutively activated RAS is also now recognized to suppress the body’s natural defense mechanisms of immune surveillance then contribute to tumorigenesis.^39^

As previously reported, polymorphisms in A-kinase anchoring proteins (AKAPs), which are components of signal transduction involving the second-messenger cAMP (Cyclic adenosine monophosphate), contribute to carcinogenesis.^40^ *APAK9* (A-kinase anchoring protein 9) binds Ser/Thr protein kinase A (PKA) and promotes PKA activation, which is a hallmark of majority tumors. PKA activation in turn activates effectors along the RAS/RAF/MAPK pathway and, as a result, is associated with poor prognosis in several tumor types.^40^ Thus, by examining late events in evolutionary trajectories in the post-administration samples, we detected that carcinogenic RAS signaling was activated, which apparently might lead to the development of acquired resistance to cetuximab.

### Transcriptional dynamic analysis of acquired resistance to cetuximab based on HNSCC PCT

**Fig. 6:**
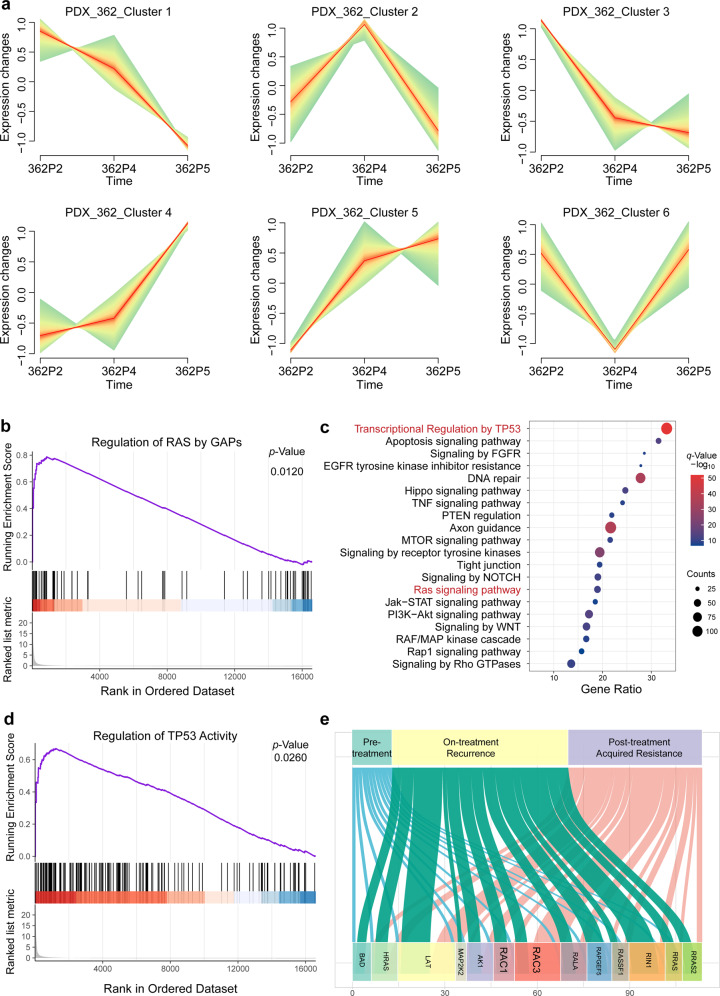
Transcriptional dynamic analysis of acquired resistance to cetuximab. **a** Clustering genes by similar expression profiles in PDX_362 for time-series samples. **b**, **d** Gene set enrichment analysis (GSEA) was performed for transcript abundance for post-treatment versus pre-treatment: the differentially expressed genes displayed enrichment for the regulation of RAS by GAPs gene sets and regulation of the TP53 activity gene sets. **c** Gene ontology (GO) analysis of differentially expressed genes in post-treatment versus pre-treatment samples. **e** Sankey diagram illustrating changes of the expression components in the RAS signaling pathway through cetuximab treatment

Our analyses of evolutionary trajectories of acquired resistance based on gene mutations and copy number variations linked mutational activation of RAS signaling to acquired cetuximab resistance. We therefore re-assessed our RNA-seq data for the acquired drug resistance cases and noted consistent trends of altered expression for genes during resistance acquisition. For example, in 3 cases involving pre-treatment, on-treatment (recurrence), and post-treatment (acquired resistance) samples, we found that 317 genes showed a trend of increased expression in at least two samples and that 389 genes showed decreased expression in at least two samples (Fig. 6a and Supplementary Fig. 9a–d). A subsequent gene ontology (GO) analysis indicated activation of the RAS signaling pathway, transcription regulation by TP53, and EGFR tyrosine kinase inhibition among the genes whose expression continued to increase as cetuximab resistance was acquired (Fig. 6c).

More broadly, GSEA indicated that the differentially expressed genes are enriched for functions related to RAS regulation by GAPs and regulation of TP53 activity (Fig. 6b, d). These data again support that RAS signaling activity is elevated as cetuximab resistance is acquired. Together, using longitudinal genomic and transcriptomic analyses of cases in the acquired resistance group, we have detailed the major evolutionary trajectories under cetuximab therapy.

### Therapeutic intervention in preclinical trials to overcome acquired resistance to cetuximab in HNSCCs

**Fig. 7:**
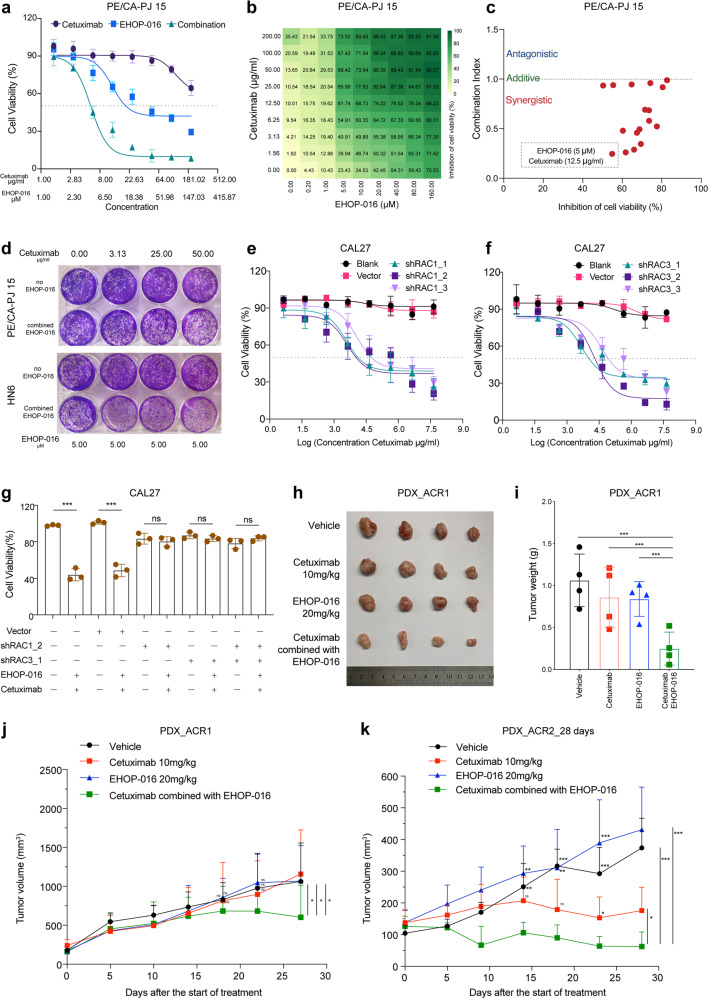
Therapeutic intervention of RAC1 and RAC3 by EHOP-016 to overcome resistance to cetuximab in HNSCCs. **a** Dose–response effects of cetuximab, EHOP-016 alone or in combination on the inhibition of cell viability in PE/CA-PJ 15 cells estimated after 3 days of drug treatment using CCK-8 assays. Each condition represents at least three biological replicates. **b** The 9 × 9 heatmaps for the combination of cetuximab with EHOP-016 in PE/CA-PJ 15 cells. **c** Combination index (CI) scores of EHOP-016 and cetuximab combination in PE/CA-PJ 15 cells, calculated at various effect levels (bottom). **d** Colony-formation assays performed with PE/CA-PJ 15 cells and HN6 cells, upon treatment with the RAC1/RAC3-inhibitor EHOP-016 alone, cetuximab alone, or a combination treatment comprising EHOP-016 and cetuximab at the indicated concentrations. **e** Cell viability was determined in CAL27 cells transfected with blank (no virus), *RAC1* shRNA-expressing lentivirus (shRAC1_1, shRAC1_2, shRAC1_3) and empty vector lentivirus (vector) in the presence of dose gradients of cetuximab using a CCK8 assay. **f** Cell viability was determined in CAL27 cells transfected with blank (no virus), *RAC3* shRNA-expressing lentivirus (shRAC3_1, shRAC3_2, shRAC3_3) and empty vector lentivirus (vector) in the presence of dose gradients of cetuximab using a CCK8 assay. **g** Cell viability was determined in CAL27 cells transfected with blank (no virus), *RAC1* shRNA-expressing lentivirus (shRAC1_2), *RAC3* shRNA-expressing lentivirus (shRAC3_1), and empty vector lentivirus (vector) with or without the combination treatment of EHOP-016 (5 µM) and cetuximab (12.5 µg/ml) using a CCK8 assay. **h**–**k** Three weeks after the start of treatment, tumor growth curves in the cetuximab-acquired resistance PDX model (PDX_ACR1, PDX_ACR2) treated with solvent control (vehicle), EHOP-016, and cetuximab, alone or in combination (as indicated). Tumor growth curves of PDX_ACR1 (**j**) and PDX_ACR2 (**k**). Photographs of tumors (**h**). Tumor weight of PDX_ACR1 (**i**). *n* = 4 mice for each group in PDX_ACR1, *n* = 5 mice for each group in PDX_ACR2. Mean tumor volumes ± SD are plotted. *P ≤ 0.05; **P ≤ 0.01; ***P ≤ 0.001

The expression of the RAS signaling components *RAC1* and *RAC3* was significantly increased in the recurrent and acquired resistance samples (Fig. 6e). Building on this insight, we speculated that blocking the activation of RAS signaling could help to overcome cetuximab resistance. We pursued this potential intervention with experiments using the small molecule EHOP-016,^41,42^ which is known to target and inhibit both RAC1 and RAC3, impeding the activity of the RAC downstream effector p21-activated kinase and cell migration in cancer cells.^42^ The HNSCC cell lines PE/CA-PJ 15 and HN6 are known to be resistant to cetuximab,^43^ and we confirmed as follows: at a cetuximab concentration of 50 µg/ml, only 13.65% and 12.64% of cells displayed proliferation defects (Fig. 7b and Supplementary Fig. 10b).

To investigate the potential of blocking RAS signaling overcome the acquired resistance of cetuximab in HNSCCs, we conducted cell proliferation experiments with two HNSCC cell lines (PE/CA-PJ 15 and HN6) using different concentration gradients of cetuximab (0, 1.56, 3.13, 6.25, 12.50, 50.00, 100.00, 200.00 µg/ml), EHOP-016 (0, 1.25, 2.50, 5.00, 10.00, 20.00, 40.00, 80.00, 160.00 µM) and the combination of the two corresponding concentrations (Fig. 7a and Supplementary Fig. 10a). Cetuximab monotherapy exhibited almost no cell proliferation inhibition (with the IC50 values greater than 200.00 µg/ml in the two cells), and EHOP-016 monotherapy also showed poor cell proliferation inhibition (with an IC50 of 23.76 µM in PE/CA-PJ 15 cells and 65.98 in µM in HN6 cells). In contrast, EHOP-016 can improve the drug sensitivity of cetuximab in two HNSCC cell lines. When inhibiting 50% cancer cell proliferation, in PE/CA-PJ 15 cells, the cetuximab concentration was 6.86 µg/ml and EHOP-016 was 5.49 µM (Fig. 7a); in HN6 cells, the cetuximab concentration was 7.44 µg/ml and EHOP-016 was 5.96 µM (Supplementary Fig. 10b).

We then mixed serial dilutions of cetuximab and EHOP-016 in all combinations (9 × 9 dose matrix) and measured the effects on cell viability after 72 h (Fig. 7b and Supplementary Fig. 10b). Our assessment of apparent synergy was based on the Loewe additivity–based combination index (CI) score.^44^ We calculated CI scores for each drug combination on the basis of half-maximal effective concentration (EC_50_) values obtained from sigmoid-fitted dose–response curves (Fig. 7b, c and Supplementary Fig. 10b, c). CI scores less than 0.75 are considered to be synergistic, scores larger than 1.5 are considered to be antagonistic, and the remainder is considered to be additive.^44^ In contrast to the failure of EHOP-016 or cetuximab monotherapy in inhibiting the proliferation of HNSCC cells, the tested combinations of these drugs were synergistic in both cell lines. Very briefly, the strongest synergistic effect for inhibiting HNSCC cell viability was detected for the combination comprising 12.5 µg/ml cetuximab with 5 µM EHOP-016 in PE/CA-PJ 15 cells (Fig. 7b, c), and 12.5 µg/ml cetuximab with 10 µM EHOP-016 in HN6 cells (Supplementary Fig. 10b, c). Furthermore, with a 14-day colony-formation assay, the two HNSCC cell lines were treated with various doses of cetuximab (0, 3.13, 25.00, 50.00 µg/ml) and a single concentration of EHOP-016 (5 μM). Results also showed that co-exposure to EHOP-016 could significantly overcome the resistance of these cells to cetuximab (Fig. 7d).

To confirm blocking RAC1/RAC3 activity increases cetuximab sensitivity with EHOP-016 is not an off-target (RAC1/RAC3 non-specific) pharmacological effect, we utilized short hairpin RNA (shRNA)-expressing lentivirus to knockdown *RAC1* or *RAC3* expression in HNSCC cells and detected whether cells’ sensitivity to cetuximab increased. Firstly, we performed qPCR to detect *RAC1* and *RAC3* expressions in 13 HNSCC cell lines: most of these HNSCC cell lines had high *RAC1* and *RAC3* expressions (Supplementary Fig. 10d, e). We then selected CAL27 cells, which have relatively high levels of both *RAC1* and *RAC3* expression, for subsequent validation (Supplementary Fig. 10d, e). To achieve a specific knockdown of *RAC1*, CAL27 cells were transfected with a *RAC1* shRNA-expressing lentivirus. Western blotting (with quantification) showed that the RAC1 level was significantly lower in the *RAC1*-knockdown cells as compared to the empty vector control group (Supplementary Fig. 10f), and knockdown was also supported by qPCR data (Supplementary Fig. 10g). Using the same approach, we knocked down *RAC3* expression in CAL27 cells (Supplementary Fig. 10f, g).

Then, we conducted cell proliferation experiments using different concentration gradients of cetuximab in *RAC1*-knockdown cells, *RAC3*-knockdown cells, the empty vector-transfected cells, and the CaL27 cells. We observed increased cetuximab sensitivity with *RAC1* silencing or *RAC3* silencing in CAL27 cells (Fig. 7e, f). Finally, we performed a combined EHOP-016 (5 µM) and cetuximab (12.5 µg/ml) treatment in multiple groups of cells (*RAC1*-knockdown cells, *RAC3*-knockdown cells, *RAC1/RAC3*-knockdown cells, the empty vector-transfected cells, and the CAL27 cells). We only found increased sensitivity to cetuximab in the presence of the RAC1/RAC3-inhibitor EHOP-016 in the empty vector-transfected cells and the CAL27 cells, indicating that the combined effects of EHOP-016 and cetuximab is really related to the target RAC1/RAC3 in RAS signaling pathway (Fig. 7g).

We used PDXs of acquired resistant cases to assess the in vivo therapeutic effects of the EHOP-016/cetuximab combination to determine whether blocking RAS signaling helps to overcome acquired resistance. The PDX_ACR1 and PDX_ACR2 models were induced from two acquired cetuximab resistance cases (PDX_419 and PDX_500, respectively) in the first PCT from two different patients. Three weeks after the second round of treatment started, PDX_419 and PDX_500 were classified as mPD cases, and were placed in the acquired resistance group. When the tumor volume of acquired resistant cases exceeded 500 mm^3^, we sacrificed the mice to obtain tumor tissues for preservation and passage (defined as PDX_ACR1 and PDX_ACR2). Two cases of acquired resistant PDXs were treated with EHOP-016 or cetuximab alone or in combination: the combined therapy exerted significant tumor suppression effects 3 weeks after treatment initiation (Fig. 7h–k and Supplementary Fig. 10h–j). Examining the PDX_ACR2 model specifically, the combined therapy achieved complete tumor remission (mCR), whereas the treatment of EHOP-016 monotherapy exerted no therapeutic effect; moreover, the tumor also progressed in the cetuximab monotherapy group (Fig. 7k).

**Fig. 8:**
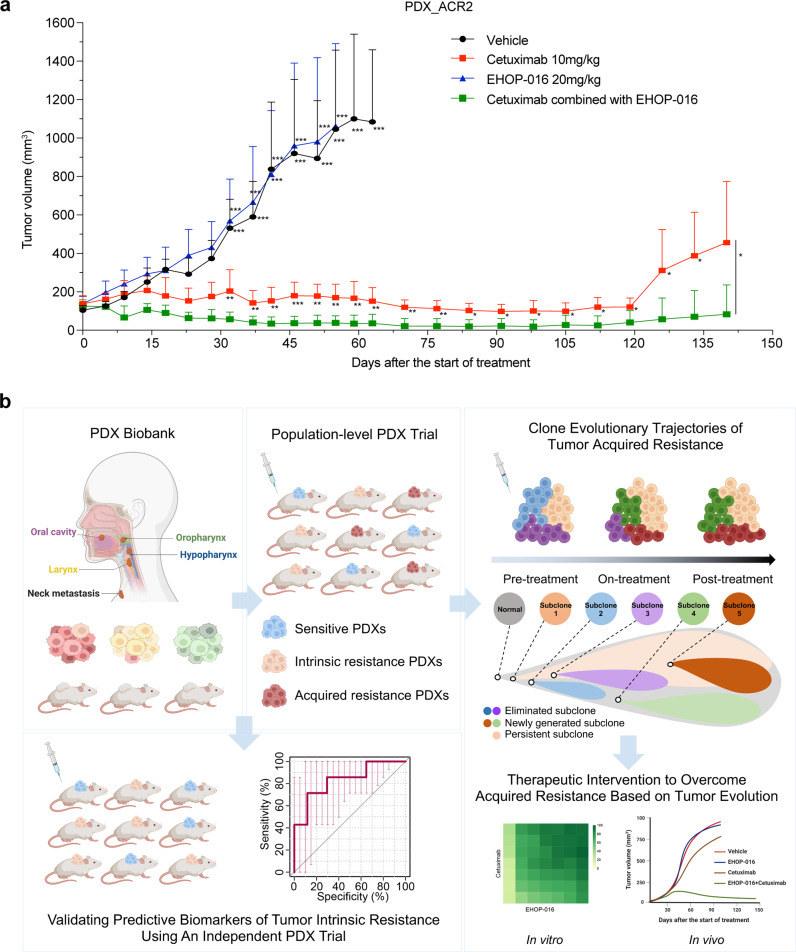
Long-term combined treatment overcame acquired resistance to cetuximab and inhibited tumor recurrence. **a** More than 4 months after the start of treatment, the tumor growth curves in the cetuximab-acquired resistance PDX model (PDX_ACR2) treated with solvent control, EHOP-016, and cetuximab, alone or in combination. *n* = 5 mice for each group. Mean tumor volumes ± SD are plotted. *P ≤ 0.05; **P ≤ 0.01; ***P ≤ 0.001. **b** Schematic diagram of the clinical utility of PDX cohorts to reveal biomarkers of intrinsic resistance and clonal dynamics underlying acquired resistance to cetuximab in HNSCCs. Schematics were created with BioRender.com

To test the long-term combined treatment efficiency in the PDX_ACR2 model, we extended the treatment time to 60 days, when all mice in the combined treatment group achieved mCR. We then measured the tumor volume and body weight of the mice over two months in the cetuximab group and the combination group to monitor tumor recurrence in the two groups. The results showed that blocking the RAS pathway not only improved the sensitivity to cetuximab in the acquired resistant model but also inhibited tumor recurrence (Fig. 8a and Supplementary Fig. 10j). Collectively, these in vitro and in vivo results support that a combination therapy comprising the RAC1 and RAC3 dual-inhibitor EHOP-016 plus cetuximab is an effective regimen for treating cetuximab-resistant HNSCCs.

## Discussion

The present study used a large-scale HNSCC PDX model platform and demonstrated an approach that enables the identification of predictive biomarkers of intrinsic drug resistance and that supports research into the varied mechanisms underlying acquired drug resistance. We conducted a randomized PCT to evaluate cetuximab monotherapy against HNSCCs at the population level. Beyond confirming that our PCT cohort informatively reproduced previously reported clinical responses to cetuximab, we found that the PDXs also informatively represent the clinical efficacy of cetuximab in the corresponding patients. We also discovered several predictive biomarkers of intrinsic drug response—including the gain of *CDK1, ANKH*, and *HEPHL1*, as well as overexpression of *MAPK15, SIX2*, and *PARP3*—and we conducted an independent PCT study with 61 PDXs that validated these biomarkers. Furthermore, we mapped the evolutionary trajectories in acquired resistance to cetuximab and detected activation (both mutational and transcriptional) of RAS signaling during the acquisition of resistance. Our functional insights motivated us to examine an ultimately successful combination therapy comprising cetuximab and the dual-target inhibitor EHOP-016, which suppressed tumor growth in all cetuximab-acquired resistant mice, thereby revealing a promising therapeutic strategy for this highly recalcitrant carcinoma (Fig. 8b).

To our knowledge, the present study is the first PCT study with population-level PDX models in HNSCCs aimed at discovering and verifying cetuximab biomarkers and elucidating the mechanisms of acquired resistance. PCT studies rely on large-scale PDX model biobanks.^15,18,19,45^ To date, PCT research has not been fully applied to preclinical compound efficacy evaluation or other translational medicine research.^14^ Recently, a key feature that has driven the development of PCT research is the ability of PDXs to predict drug potency and resistance in patient tumors.^46^ Based on this advantage, randomized phase II-like studies of PDXs in mice are applicable for assessing a range of therapeutic agents. Another major advantage of PDX tumor models driving the development of PCT studies is their capability of capturing therapy-induced cancer progression.

PCT studies of CRCs have validated *KRAS* mutation as an informative biomarker for cetuximab resistance;^21,47^ these findings have actually supported revision of the NCCN guidelines for use of cetuximab for CRCs.^48^ In addition, several PCT studies revealed that mutations in genes including *NRAS, BRAF, FGFR1*, and *PIK3CA* (among others) are potential biomarkers for intrinsic resistance to cetuximab in CRCs.^8,17,21,49,50^ Notably, none of these biomarkers found in CRC PCTs were detected in our HNSCC PCT. *KRAS, BRAF*, and *NRAS* were wild-type in our PCT, and we detected no relationship between these genes and cetuximab resistance in HNSCCs. For *PIK3CA*, we detected a mutation in 1 case in the intrinsic resistance group (1 of 8), detected amplification in 2 sensitive cases (2 of 7) and 2 acquired resistance cases (2 of 5). These inconsistent observations regarding mutations and copy number variations do not support a robust association between *PIK3CA* and cetuximab resistance in HNSCCs.

Of particular note, our findings regarding the impacts of *TP53* mutation on cetuximab efficacy in HNSCCs are opposite to previously reported conclusions about CRCs, which again likely reflect differences in tumor types. That is, several clinical studies of CRCs have reported that *TP53* mutation is associated with a poor prognosis for patients treated with cetuximab.^48,51–54^ Another CRC study suggested that *TP53* mutations are predictive for cetuximab sensitivity, especially for patients without *KRAS* mutations,^55^ and proposed that *TP53* genotyping could be useful in the clinic to help select CRC patients who are likely to benefit from cetuximab-based treatment. Thota et al. (2021) found that *APC* and *TP53* mutations can predict cetuximab sensitivity across consensus molecular subtypes, based on an analysis of multiple CRC tumor/PDX/cell line datasets.^56^ In our HNSCC PCT, all of the PDXs were wild-type *KRAS*, and we found that *TP53* mutation was related to the sensitivity of cetuximab: the mutation rate of *TP53* in the sensitive group was 100% but was only 62.5% in the intrinsic resistance group.

The predictive biomarkers presented here demonstrate the utility of performing RNA-seq and WES on tumor tissues obtained longitudinally at multiple time points during targeted therapy in PDXs of advanced solid malignancies. Previous studies have shown that the intraocular homologous box (SIX) transcription factor *SIX2* promotes breast cancer metastasis via transcriptional and epigenetic control of E-cadherin expression;^57^ the *SIX2* level is correlated with poor prognosis, and elevated *SIX2* has been shown to decrease the sensitivity of hepatocellular carcinoma cells to 5-FU.^58^ Wu et al. (2017) found that *DDX3*-mediated cetuximab resistance can be regulated by the YAP1/SIX2 axis in *KRAS*-WT CRCs,^59^ and further confirmed that *SIX2* had prognostic value (albeit only in *KRAS*-WT CRC patients).^57–59^ These findings from previous studies support the utility of the predictive biomarkers we identified with the HNSCC PCTs. We also found several previously unknown predictive biomarkers, including Kruppel like factor 9 (*KLF9*),^60^ Poly (ADP-Ribose) polymerase family member 3 (*PARP3*),^61^ and Podocalyxin like 2 (*PODXL2*);^62^ none of these have been previously associated with tumor drug resistance or reported as predictive biomarkers for targeted therapies. Beyond this, we could utilize machine learning to derive multi-gene expression signatures that predict drug responses to specific treatments.^63^

Resistance to therapy could be innate or acquired.^64^ Multiple studies have demonstrated the importance of clonal diversity and dynamics in resistance to therapeutic agents,^65^ and the use of model systems like PTCs can be extremely useful in supporting clonal expansion studies about the process of acquired resistance.^46^ Consider that in a typical clinical setting, large-scale molecular profiling of a patient’s tumor can only be conducted for a few time points, i.e., when the tumor is surgically removed or when a biopsy is taken. This limited access to patient tumor samples severely curtails our ability to investigate the mechanisms underlying the malignant progression of a patient’s cancer.^34^ In contrast, the use of PDX tumor models allows frequent examination of PDX tissue specimens (for example as a function of time), thus providing exceptional opportunities to investigate the molecular and cellular mechanisms that underlie tumor progression and therapy resistance.^14,46^ In addition, clinical patients often take a variety of drugs, which results in complex forms of drug resistance (often via multiple interference factors;^66^) thus, PDX clinical trials represent a good preclinical model for single-drug resistance studies.

Our clonal dynamic work in the present study indicated that acquired resistance to cetuximab apparently results largely from (i) the acquisition of a diverse set of newly generated subclones and (ii) the elimination of sensitive subclones. For instance, we observed an association between acquired resistance and mutational activation of mutant *MMP3* (PDX_563, CCF increased from 0 to 6.13%) and mutant *MMP13* (PDX_549, CCF prevalence increased from 0 to 11.60%). One trend was particularly noteworthy: amplification of *MMP10* was detected post-treatment in 40% (relative to their matched pre-treatment samples). Beyond indicating that a wider scope of matrix metalloproteinase (MMP) family members could represent informative biomarkers for cetuximab response, these findings support the idea of targeting MMPs as an intervention to overcome cetuximab resistance. Activation of the MMP family was confirmed based on our transcriptome analysis of the acquired resistance group and our GSEA data. Indeed, this GSEA identified “extracellular matrix” as the significantly upregulated gene set in the acquired cetuximab resistance samples. Johansson et al. reported that extracellular matrix and cancer-associated fibroblasts (CAFs) induced MMP–mediated cetuximab resistance in HNSCCs and suggested that several MMPs could cooperate to promote cetuximab resistance.^67^ This funding is consistent with our findings, which collectively support that (i) MMPs should be explored as potential cetuximab resistance biomarkers and (ii) inhibiting MMPs could improve the effects of EGFR-targeted therapies.

Another notable finding of our study was that the emergence of acquired resistance can be accompanied by a loss of subclones bearing amplification of *EGFR, CCND1*, and *FGF3*. *EGFR* amplification could represent a single predictive biomarker for cetuximab response in HNSCCs.^6^ A previous study of breast cancer reported that *FGF3* amplification is correlated with a lower pathologic complete response in patients treated with neoadjuvant anti-HER2 therapy.^68^ In 2013, Aro et al. reported that *FGF3/FGF4* amplification was frequently observed in responders to sorafenib.^69^ These studies suggest that *FGF3* amplification could improve outcomes for HNSCC cases treated with cetuximab. However, it should be noted that several studies have reported associations of *CCND1* amplification and overexpression with poor prognosis, cisplatin resistance, and EGFR-inhibitor resistance.^70^ Clearly, the impacts of *FGF3* or *CCND1* on the development of cetuximab resistance require further study.

Our study illustrates the advantages of using PDX models as living biobanks to obtain samples at different time points during the development of drug resistance, providing abundant sample materials to support the discovery of biomarkers and to conduct experimental studies of both intrinsic drug resistance and the use of drug combination strategies to combat acquired drug resistance. Looking forward, it should be informative to collect biopsy tissues and blood from patients treated with cetuximab and to conduct single-cell sequencing to help validate the findings from our PCT study.

## Materials and methods

### HNSCC tumor samples

All patients’ tumor tissues, adjacent normal tissues, and blood samples were obtained from the Ninth People’s Hospital of the Shanghai JiaoTong University School of Medicine. This study complied with the Declaration of Helsinki and was approved by the Ethics Committee of Shanghai Ninth People’s Hospital. Written informed consent was obtained from all study participants prior to the study. Immediately after surgery, two fragments were snap-frozen in liquid nitrogen for molecular characterization, two fragments were fixed in 10% neutral buffered formalin for histopathologic analysis, and the remaining fragments were transferred to culture medium for engraftment.

### PDX model generation

All animal experimental procedures were approved by the Institutional Animal Care and Use Committee of the Ninth People’s Hospital of the Shanghai JiaoTong University School of Medicine. Fresh tissue was fragmented and either frozen or prepared for implantation. After patient surgery, the tumor samples were engrafted onto 2–3 nude mice. Small fragments (50 mm^3^) were subcutaneously engrafted into the scapular area of anesthetized mice; the grafted tumor was the P0 generation. Tumor volumes (TVs) were determined by the formula: TV = (width^2^ × length) × 0.5. Tumor growth was measured at least once a week using a digital caliper and serial fragment grafts of each tumor were conducted on three nude mice for the P1 generation (when the tumors reached a volume of 800–1500 mm^3^). The remaining tumor grafts were fresh-frozen in liquid nitrogen for molecular analyses or fixed in 10% neutral buffered formalin for pathologic analyses.

### Cell viability assays

Cells were seeded in 96-well plates at densities that resulted in near-confluency after 72 h of drug treatment. After 24 h, the media was aspirated and 100 μL of fresh media was added (containing different concentrations of cetuximab, EHOP-016, or a combination) in technical triplicates. After 72 h of treatment, 100 μL fresh CCK-8 diluted 1:10 with media was added to each well, and the cells were incubated at 37 °C for 2 h. After incubation, optical density values were determined at a wavelength of 450 nm on a Biotek Microplate Reader.

### Statistical analysis

GSEA v4.1.0, GraphPad PRISM 8, and R 3.6.3 were used for the statistical analyses. The specific statistical tests used are specified in the figure legends. Error bars, SD, unless otherwise stated. The threshold for statistical significance was *P* ≤ 0.05, unless otherwise specified.

## Supplementary information

Supplementary Materials

## Data Availability

All data associated with this study are present in the paper or the Supplementary Materials. Any other relevant data are available from the corresponding author upon reasonable request.
