# Supplementary material for: Clinical utility of PDX cohorts to reveal biomarkers of intrinsic resistance and clonal architecture changes underlying acquired resistance to cetuximab in HNSCC
Source: Signal Transduct Target Ther. 2022 Mar 8;7:73. doi: 10.1038/s41392-022-00908-0 (PMC8904860; doi:10.1038/s41392-022-00908-0)
Supplement: Supplementary file 1 — Supplementary Materials [file 41392_2022_908_MOESM1_ESM.docx]

Supplementary Materials for

**Clinical Utility of PDX Cohorts to Reveal Biomarkers of Intrinsic Resistance and Clonal Architecture Changes Underlying Acquired Resistance to Cetuximab in HNSCC**

Yanli Yao^1,2#^, Yujue Wang^1,2#^, Lan Chen^1,2#^, Zhen Tian^2,3^, Guizhu Yang^1,2^, Rui Wang^1,2^, Chong Wang^1,2^, Qi Wu^1,2^, Yaping Wu^1,2^, Jiamin Gao^1,2^, Xindan Kang^1,2^, Shengzhong Duan^2,4^, Zhiyuan Zhang^1,2*^, Shuyang Sun^1,2*^.

Correspondence to: sunshuyang@sjtu.edu.cn (S. S.); zhzhy@sjtu.edu.cn (Z. Z.)

**This PDF file includes:**

Materials and Methods

Supplementary Text

Figures. S1 to S10

Tables S1 to S6

Materials and Methods

**PDX Clinical Trial**

A PDX model cohort representing 49 patients (with P2-P4 generation models) was randomly selected. After the tumor volume reached approximately 100-200mm^3^, each PDX case was randomly divided into the vehicle arm (*n* = 3) or the cetuximab monotherapy arm (*n* = 3). In the first-round treatment, PDXs were treated with vehicle (solvent control, intraperitoneal injection, twice a week) or cetuximab at a concentration of 10 mg/kg twice a week intraperitoneally for three weeks. Tumor volume (TV) and body weight measurements for mice were taken twice weekly with a digital caliper and electronic scale, respectively. The first evaluation was scheduled 21 days after treatment initiation or fast-growing tumors increased to 1000-1500mm^3^ within 21 days.

For assessment of the tumor response to cetuximab, we used volume measurements normalized to the tumor volume at the time of initiation (Day0) of cetuximab treatment. Cases were classified as follows: (1) tumor complete regression (mCR) with a decrease of at least 40% in tumor volume; (2) tumor partial regression (mPR) with a decrease of 20% in tumor volume; (3) disease progression (mPD) with at least a 30% increase in tumor volume, and (4) disease stabilization (mSD) with a tumor graft volume at levels between 30% growth and 20% regression.

After the first-round treatment initiation, in 21 days, mPD cases were identified as intrinsic resistant cases. The TV and body weight of other cases were measured twice a week up to 90 days. At 90 days, mCR cases were defined as the sensitive group. For cases that relapsed within 90 days or did not achieve mCR until 90 days, a second round of treatment was performed (same concentration and treatment time as the first round). Three weeks after the second round of treatment started, mPD cases were defined as the acquired resistance group. Cases where recurrence occurred in the first-round treatment but mPR or mCR occurred after the second round of treatment, indicated that cancer cells entered a reversible drug-tolerant persister (DTP) state to evade death from targeted agents. In the PCT, humane endpoints to euthanize mice were tumor volumes greater than 2000 mm^3^, loss of > 20% body weight, and ulceration or severe necrosis of tumor.

**Whole-exome Sequencing**

The Illumina NovaSeq 6000 platform (Illumina, San Diego, USA) was utilized for whole-exome sequencing at Novogene Bioinformatics Technology Co., Ltd (Beijing, China) to generate 150-bp paired-end reads with a minimum coverage of ~99% of the genome. The average depth was 200× in the patient tumor samples and PDX samples, and 100× in the normal samples. As PDX specimens include both human (tumor) and mouse (stromal) origins, the sequencing reads were mapped to a hybrid human and mouse genome (hs37d5 and mm10) via Burrows-Wheelers Aligner (BWA) to separate human and mouse reads. Human variants were called with SAMtools, Genome Analysis Toolkit (GATK-Unified Genotyper), and FreeBayes (Garrison and Marth). Appropriate filters were used i) to discard low-quality variants and ii) to enrich for likely functional somatic mutations by excluding silent variants and germline SNPs.

**RNA-Sequencing**

RNA was prepared with rRNA-depleted RNA by the ultra-directional RNA library prep kit for Illumina (NEB, Ipswich, USA) following the manufacturer’s recommendations. Clustering of the index-coded samples was performed on a cBot Cluster Generation System using TruSeq PE Cluster Kit v3-cBot-HS (Illumina, San Diego, USA) according to the manufacturer’s instructions. After cluster generation, the libraries were sequenced on an Illumina HiSeq platform and 150 bp paired-end reads were generated. Sequencing reads were mapped in parallel to both the mouse (mm10) and the human (hs37d5) reference genomes using Hisat2 v2.0.5. Cufflinks v2.2.1 used the results of Hisat2 alignment to assemble transcripts, estimate the abundance of these transcripts, and detect differential expression among samples.

**Biomarker Discovery and Verification with PCTs**

In the first PCT (49 cases), a series of biomarkers for cetuximab sensitivity or resistance were found by analyzing the mutated genes, copy number variations, and differentially expressed genes in the sensitive and intrinsic resistance groups, and the accuracy of the biomarkers was further evaluated in an independent validation PCT. A total of 61 cases were included in the validation PCT. The difference from the first discovery PCT is that we performed a 1 × 1 × 1 PCT (one PDX per case with one drug treatment) ^1^. Cetuximab treatment was delivered in the same way as the first PCT; we grouped the cases: mCR and mPR were the relatively sensitive group, mPD was the intrinsic resistant group, and the remaining cases were the relatively stable group. DNA and RNA were extracted from the pre-treatment PDX tissues of the 61 cases. Isolation of total DNA from tissues was conducted using a magnetic bead genomic DNA extraction kit (TIANGEN, Beijing, China) following the manufacturer's instructions. The DNA was eluted in 50 µL of distilled H_2_O and used for further analysis. Total RNA from xenograft tissue samples was isolated by using an RNAprep pure tissue kit (TIANGEN, Beijing, China) following the manufacturer's instructions. The RNA was eluted in RNase-free 50 µL H_2_O and used for further analyses. We used high-fidelity enzymes for PCR and Sanger sequencing to detect gene mutations. Copy number variations were also detected by quantitative real‐time PCR (qPCR) with genomic DNA ^2^. Gene expression was analyzed by qPCR with cDNA. The primers used in this study are shown in **Supplementary Table5**. The biomarker performance when predicting sensitive or intrinsic resistance status was assessed using ROC analyses. The AUC, and the representative best values for the sensitivity, specificity, and accuracy at an optimal cutoff point, were used for the performance measures.

**Phylogenetic Trees**

Clonal deconvolution in PyClone ^3^ served as an input to generate phylogenetic trees in CITUP (https://github.com/amcpherson/citup). The variant allele frequencies (VAFs) from each point mutation were normalized with estimated tumor purities with PyClone. The clonal frequencies were then used as input for CITUP for the joint calculation and estimation of clonal subpopulations using the optimal trees across the longitudinal timepoints from the same case. The clonal lineages were plotted with “timescape” using the CITUP tree structures and the clonal frequencies across the longitudinal timepoints for individual cases.

**Evolutionary Cluster Inference by Transfer Learning.**

REVOLVER ^4^ was used to infer repeated evolutionary trajectories in the acquired resistance group of PDX samples. This package uses the pigeonhole principle and sum rule for phylogenetic construction, and implements a maximum likelihood method to jointly fit and identify similar trajectories among samples by transfer learning. Evolutionary distances between fitted trajectories were computed and used to stratify the cohort into subgroups of tumors that harbor similar evolutionary trajectories. CCF values of mutations were obtained from PyClone analysis. While CNVs were assigned to CCF clusters (based on presence or absence in multi-regions), their CCFs were defined as the mean CCFs of a target cluster. A jackknife approach was applied to estimate the stability of clusters and trajectories.

**Colony Formation Assays**

Cells were seeded into six-well plates at 10^3^ cells per well. After 24 h, compounds were added, and the media was replaced every three days. Cells were cultured for 12 days, and colonies were fixed with 10% neutral buffered formalin, stained with 0.05% (w/v) crystal violet (25% methanol), washed, and imaged.

**Synergy Experiments**

For dose–response curves, PE/CA-PJ 15 and HN6 cells were treated with different concentrations of cetuximab in addition to fixed concentrations of EHOP-016 to determine if they had synergistic, antagonistic, or additive effects. Potential synergy between cetuximab and EHOP-016 was evaluated by calculating the combination index (CI) based on the Loewe additivity model. The CI score, CI_X_=C1/EC_X,1_+C2/EC_X,2_ measures the fractional shift between the combination doses (C1 and C2) and the single agent’s inhibitory concentration for a given level of inhibition (EC_X,1_ and EC_X, 2_). CI scores less than 0.75 were considered to be synergistic, scores larger than 1.5 were considered to be antagonistic, and the remainder were considered to be additive. For dose–response matrices, cells were treated with log-scale concentrations of each compound in 9 × 9 grids. Cell viability was measured with CCK-8 assays, and data were normalized to the average of the untreated wells. All synergy experiments were performed with technical triplicates.

**Plasmid construction and lentivirus preparation**

The shRNA lentiviral plasmid targeting RAC1 or RAC3 was constructed by inserting annealed shRNA template DNA sequence into the pLKO.1 vector. Lentivirus production was performed by transfecting viral packaging vectors pCMV-VSV-G and pΔ8.9 into 293T cells using Lipofectamine 3000 (Thermo Fisher Scientific, Waltham, USA) according to the manufacturer’s instructions. See **Supplementary Table5** for the shRNA sequences used in the study.

***In vivo* Combination Therapy Study**

Two cases of cetuximab-acquired resistant PDX models (PDX_ACR1, PDX_ACR2) were used to evaluate the effect of the combination of cetuximab and EHOP-016 *in vivo*. Each PDX case was established based on subcutaneous transplantation into 30 nude mice; TV was then measured once every three days. After the volume reached 100mm^3^-200mm^3^, 24 mice were selected according to the TV and body weight, for grouping into the vehicle group, the cetuximab monotherapy group, the EHOP-016 monotherapy group, or the combination group (4-5 mice for each group). The treatment for each group of fast-growing PDX_ACR1 was as follows twice a week for 2 weeks: solvent control, intraperitoneally; cetuximab, 10 mg/kg intraperitoneally; EHOP-016, 20 mg/kg intraperitoneally；cetuximab, 10 mg/kg combined with EHOP-016, 20 mg/kg intraperitoneally. The treatment concentration for each group of PDX_ACR2 was the same as that for the PDX_ACR1. To test the effect of long-term combined treatment in acquired resistance cases, we extended the treatment time to 60 days (twice a week), when all mice in the combined treatment group achieved mCR. We then measured the TV and body weight of the mice over two months in the cetuximab group and the combination group to monitor tumor recurrence in the two groups.

**Statistical Analysis**

GSEA v4.1.0, GraphPad PRISM 8, and R 3.6.3 were used for the statistical analyses. The specific statistical tests used are specified in the figure legends. Error bars, SD, unless otherwise stated. The threshold for statistical significance was *P ≤ 0.05*, unless otherwise specified.

**Reference**

1. Gao H*, et al.* High-throughput screening using patient-derived tumor xenografts to predict clinical trial drug response. *Nat Med.* **21**, 1318-1325 (2015).

2. Licitra L*, et al.* Evaluation of EGFR gene copy number as a predictive biomarker for the efficacy of cetuximab in combination with chemotherapy in the first-line treatment of recurrent and/or metastatic squamous cell carcinoma of the head and neck: EXTREME study. *Ann Oncol.* **22**, 1078-1087 (2011).

3. Roth A*, et al.* PyClone: statistical inference of clonal population structure in cancer. *Nat Methods.* **11**, 396-398 (2014).

4. Caravagna G*, et al.* Detecting repeated cancer evolution from multi-region tumor sequencing data. *Nat Methods.* **15**, 707-714 (2018).

Supplementary Text

**Supplementary Results**

**Establishing and Characterization of the HNSCC PDX Models Biobank**

We undertook efforts aiming to build a biobank of surgical materials from HNSCC patients, which were stored under viable conditions and serially propagated in mouse recipients. For each tumor specimen, some fragments were collected for pathologic and molecular characterization; others were subcutaneously implanted in several mice and then expanded to generate a pair of independent xenograft lines from the same patient tumor.

To elucidate genetic alterations in these carcinomas, we enriched PDXs and performed whole exome sequencing (WES) of the tumor grafts, the parental tumors, and matched normal tissues from each donor patient. Variants were identified independently, and then the union of these genetic events was re-analyzed in both samples to detect and recover variants with low sequencing coverage and/or a low variant allele fraction (VAF). To evaluate whether VAFs were conserved at the exome-wide scale, we calculated the correlation between 5 pairs of case-matched PDXs and tumors. The Pearson correlation coefficient between matched tumors and PDXs ranged from 0.3740 to 0.6860 (median for matched samples: 0.4981; median for unmatched samples: -0.0581; **Fig. S1a**). ­The WES data also confirmed a high correlation of the patients’ parental tumors and their PDXs based on *i*) the percentage of tumor variants maintained in their respective PDXs, average 97.45 % of all variants identified in primary tumors were also detected in their matched PDXs (**Fig. S1b**); and *ii*) linear regression across the VAF distributions, two representative cases show relatively high correlation in VAF distribution (R^2^ = 0.9150 and 0.8575, respectively) (**Fig. S1c-d**).

RNA sequencing was performed on case-matched PDXs and tumors; mouse-specific reads were filtered *in silico* from the xenografts before aligning reads to the human genome. Principal Component Analysis (PCA) of the RNA sequencing data indicated that the gene expression profiles of a parental tumor and its related PDX was more similar than for other patient tumors or PDXs (**Fig. S1e**). In summary, we have established and characterized a HNSCC PDX model biobank and found that these HNSCC PDXs exhibit a high degree of genomic stability and gene expression pattern similarity in comparisons with their parental tumors.


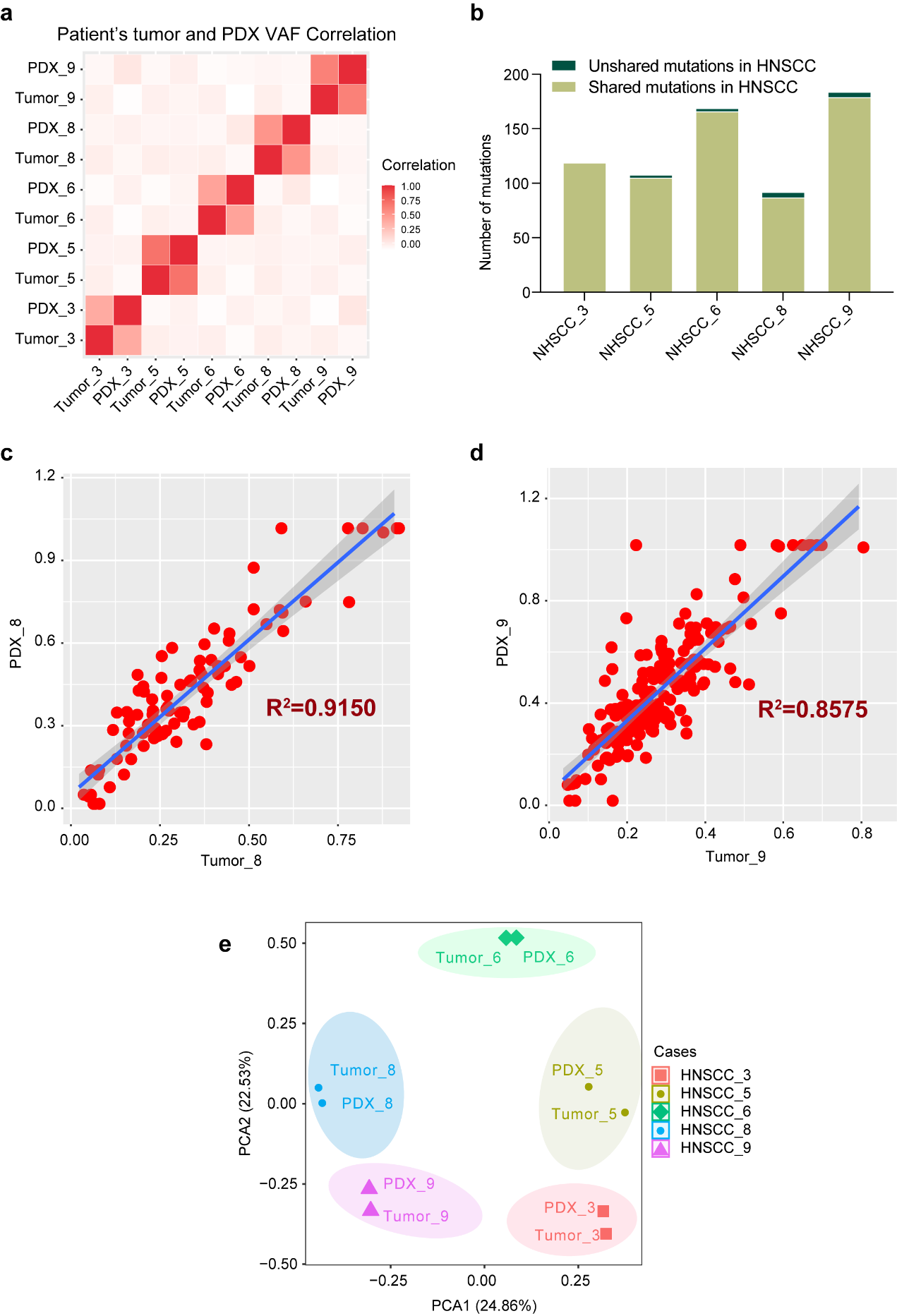


Figure. S1. HNSCC PDXs recapitulate the mutation and expression landscape of patients’ tumors

**a**. Correlation analysis of the variant allele frequencies (VAFs) in the parental tumor versus the VAFs in each PDX. **b**. The number of tumor variants maintained in their respective PDXs. **c-d**. Linear regression across the VAF distributions of two patients’ tumors and matched PDXs. **e**. RNA-seq gene expression principal component analysis (PCA) plots of parental tumors and corresponding PDXs for five HNSCC cases.


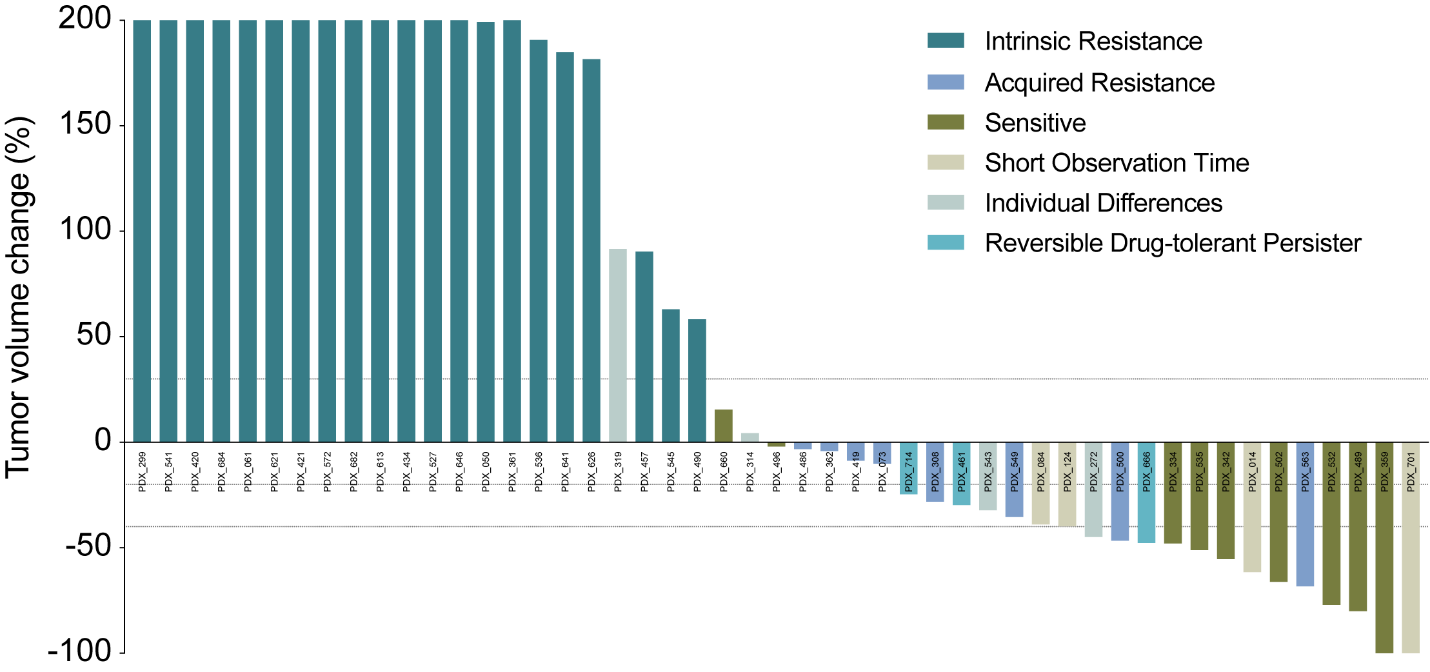


Figure. S2. Response to cetuximab in PDX models of HNSCC PCT recapitulates patients’ response related to Figure 1.

Waterfall plot representing the response of 49 cases of HNSCC PDX treated with cetuximab on 21 days after treatment started. -40%, -20%, and 30% are marked by dotted lines to indicate the range of mCR, mPR, mSD, and mPD. The mPD cases were classified as the intrinsic resistance group.


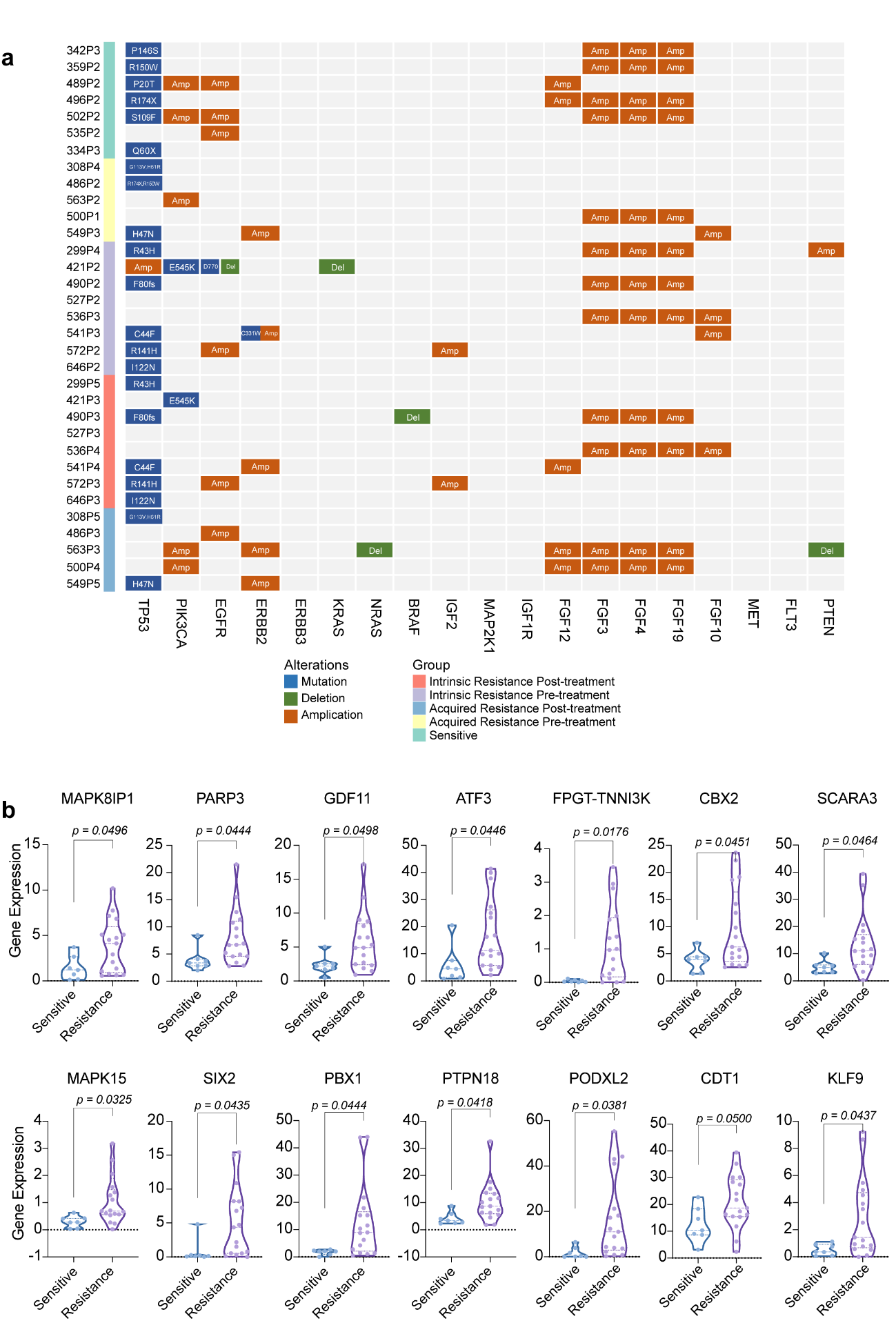


Figure. S3. Genetic and transcriptomic landscape of HNSCC PCT related to Figure 2.

**a**. Somatic mutations and amplifications/deletions in relevant genes that were previously reported related to cetuximab resistance in other tumors. Samples are displayed and indicated by color coding (green, Sensitive; yellow, Acquired Resistance pre-treatment; purple, Intrinsic Resistance pre-treatment; blue, Acquired Resistance post-treatment; red, Intrinsic Resistance post-treatment). **b**. Violin plots measure the expression level of the 14 genes which significantly differentially expressed in sensitive and resistant groups. The *P* value was calculated from a two-sided Wilcoxon rank-sum test. Mean ± SD are plotted. **P*≤0.05; ***P*≤0.01; ****P*≤0.001.


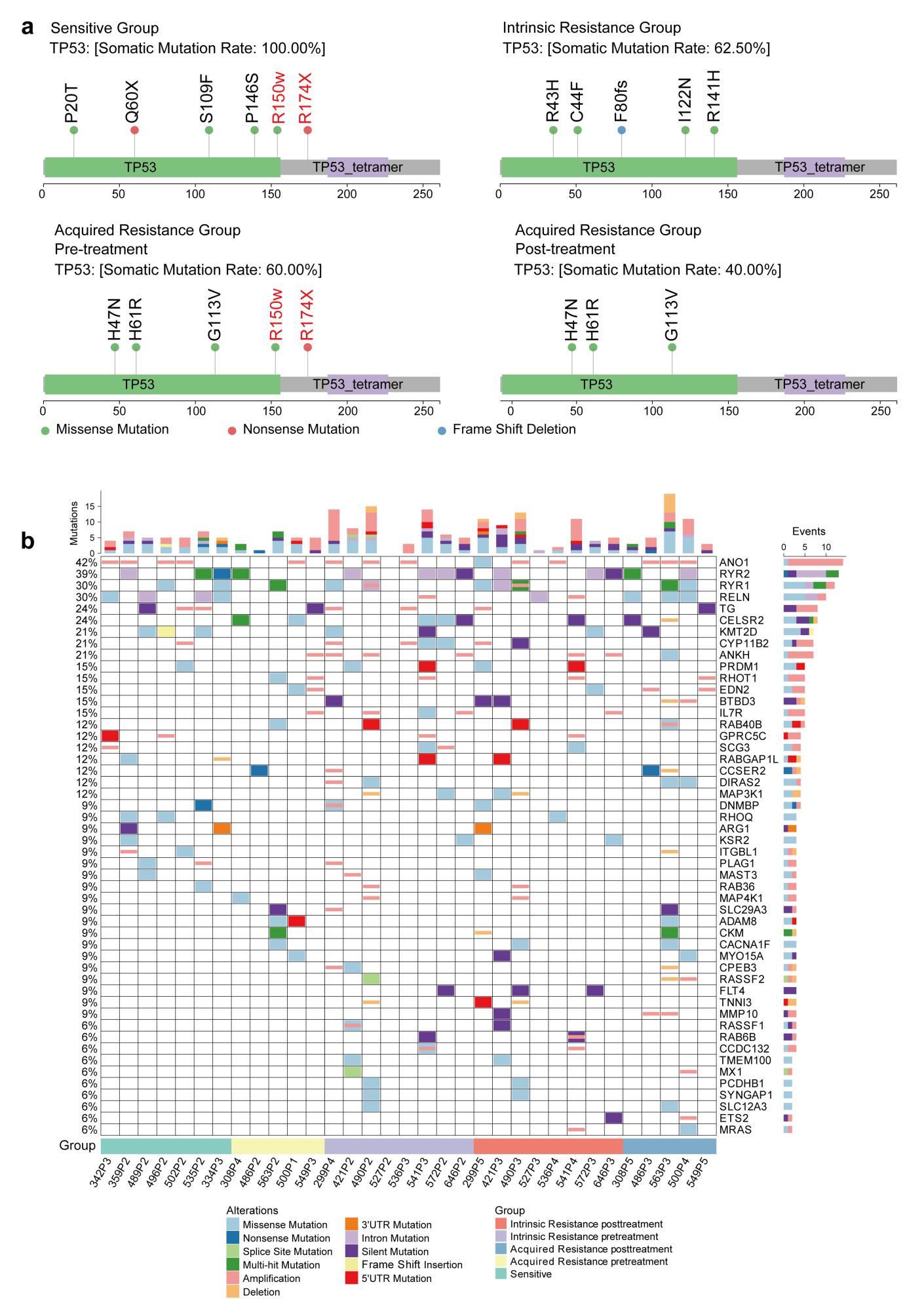


Figure. S4. Pattern of *TP53* somatic mutations and genomic alteration of RAS signaling pathways in HNSCC PCT related to Figure 2.

**a**. Lollipop plot of *TP53* mutations identified in the sensitive group, intrinsic resistance group, and acquired resistance group (pre-treatment and post-treatment). Blue, frameshift mutation; Green, missense mutation; Red, nonsense mutation. **b**. Mutations and copy number changes for select genes belonging to RAS signaling pathways are shown across HNSCC PDXs in PCT.


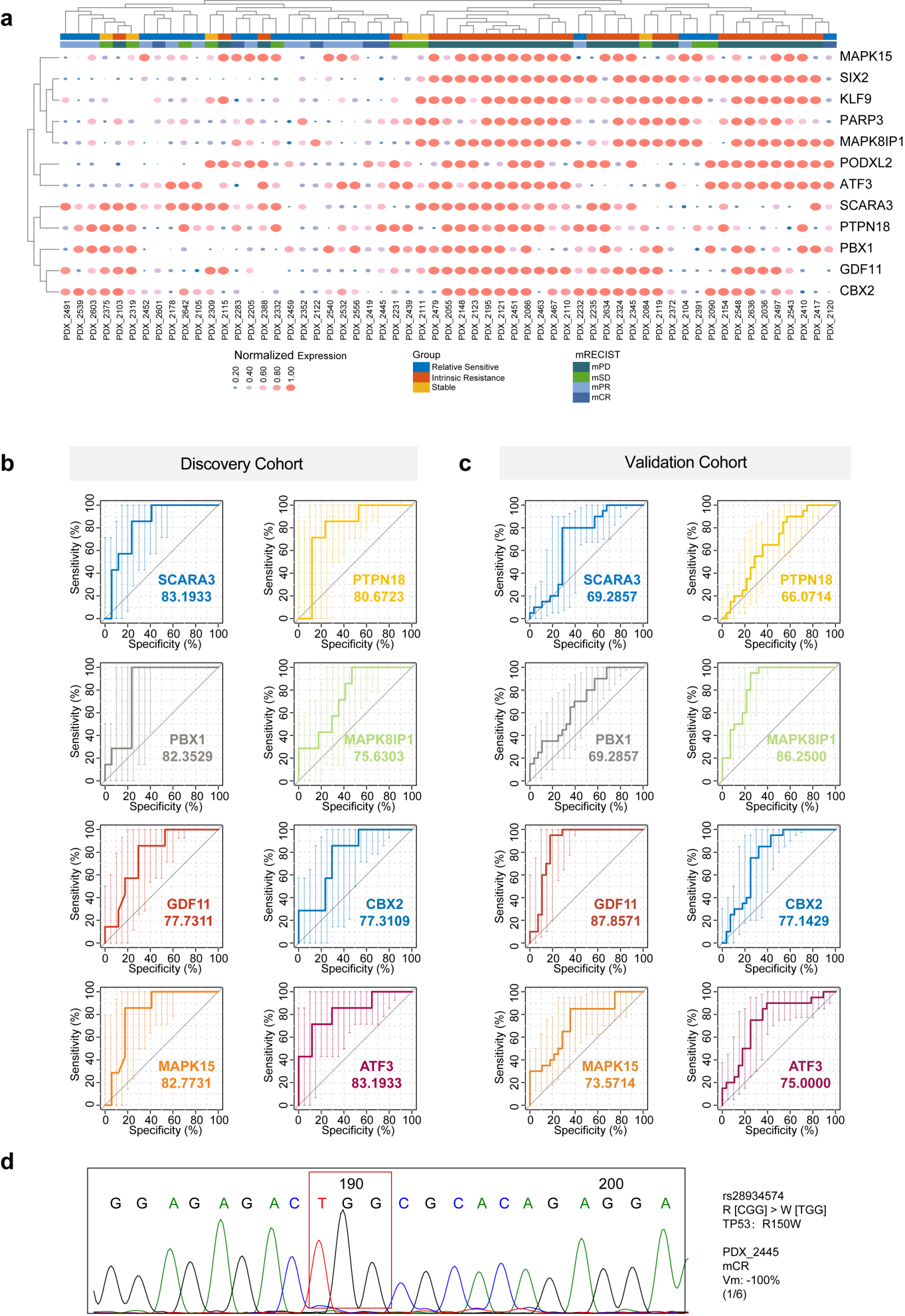


Figure. S5. Identification of predictive biomarkers for cetuximab resistance in an independent PDX cohort related to Figure 3.

**a**. Gene expression profiles of intrinsic resistance and relatively sensitive PDX samples in the validation PCT. Each sample ID is denoted by the PDX model ID. **b-c.** The ROC curves measured by RNA sequencing (**b**) and real-time qPCR (**c**) of *SCARA3, PTPN18, PBX1, MAPK8IP1, GDF11, CBX2, MAPK15* and *ATF3* in distinguishing the sensitive group and the relative resistance group. **d**. Sanger sequencing results of PCR-amplified fragments to detect *TP53* mutation (R150W and R174X) in the validation PCT.


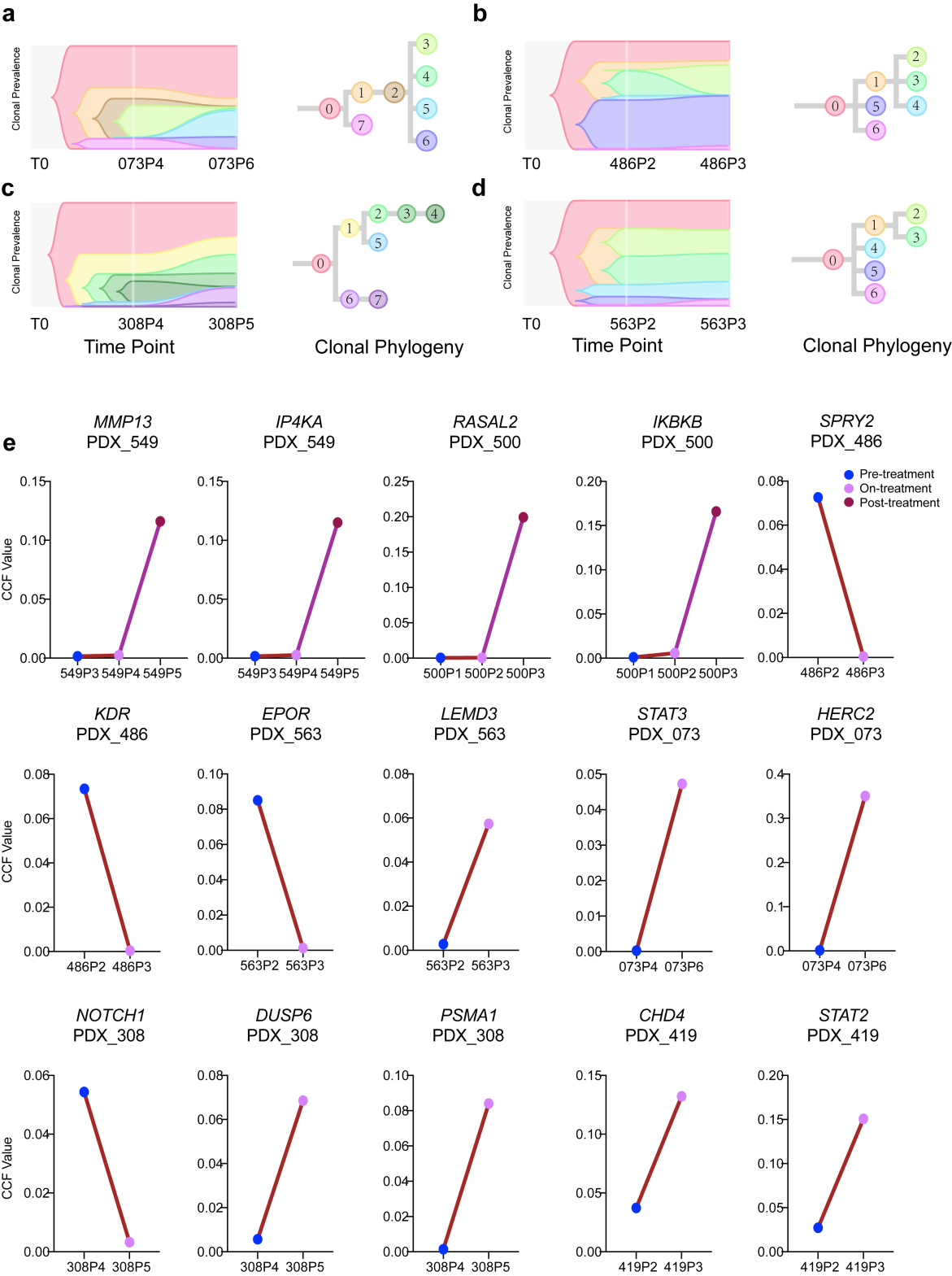


Figure. S6. Clonal structure and phylogenetic reconstruction of PDXs in the acquired resistance group related to Figure 4.

**a-d.** Clonal evolution in response to cetuximab treatment inferred from WES sequencing. Plots of clonal lineages and frequency changes over time and in response to cetuximab treatment for 4 cases (PDX_073, PDX_486, PDX_308 and PDX_563). **e.** The line graphs illustrate changes in the CCF value of 15 gene mutations through cetuximab treatment in the acquired resistance group.


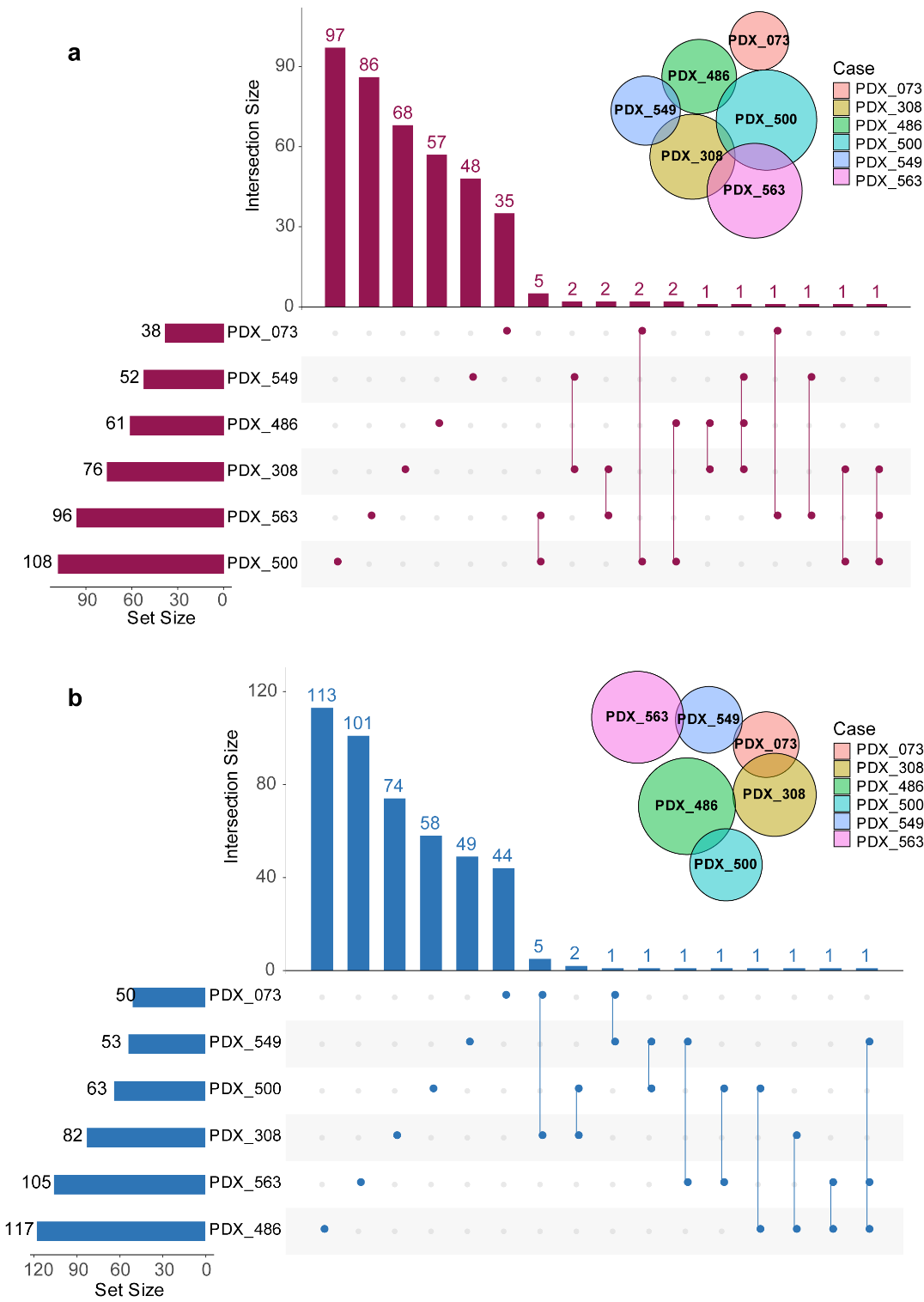


Figure. S7. Shared mutation analysis of the newly generated subclones or eliminated subclones in the acquired resistance group related to Figure 4.

**a.** Venn diagram and UpSet plot show the number of shared mutations between the newly generated subclones of 6 cases among the acquired resistance group. **b.** Venn diagram and UpSet plot show the number of shared mutations between the eliminated subclones of 6 cases among the acquired resistance group.


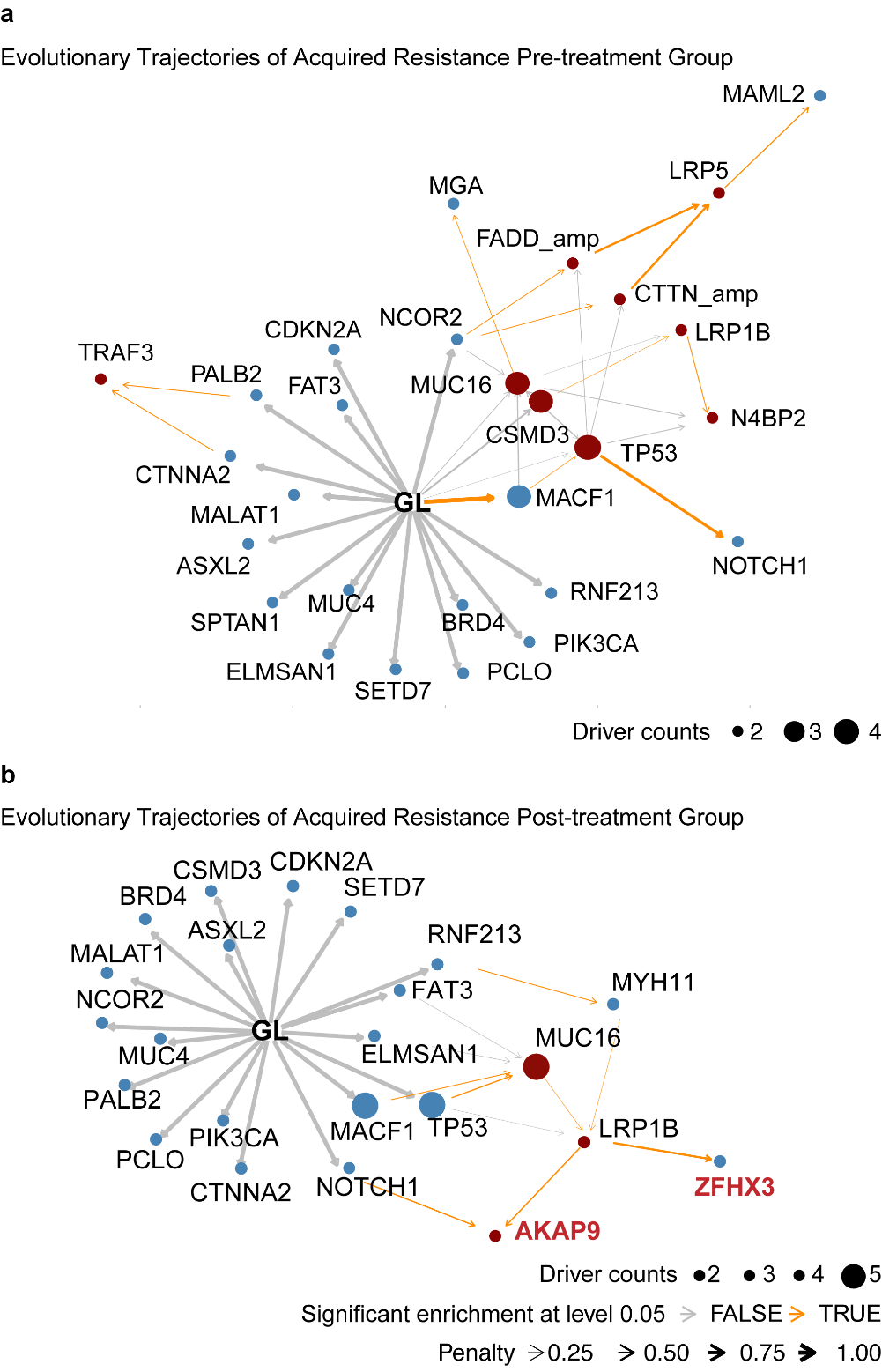


Figure. S8. Repeated evolutionary trajectories of acquired cetuximab resistance in HNSCCs related to Figure 5.

**a. b** REVOLVER analysis of PDXs with acquired cetuximab resistance. Repeated trajectories in the pre-treatment or post-treatment groups. Arrows indicate transitions. Indicated are the number of times a transition was observed among the acquired resistant samples, the number of times an alteration was clonal or subclonal in the cohort, and the probability of detecting the edge across samples.


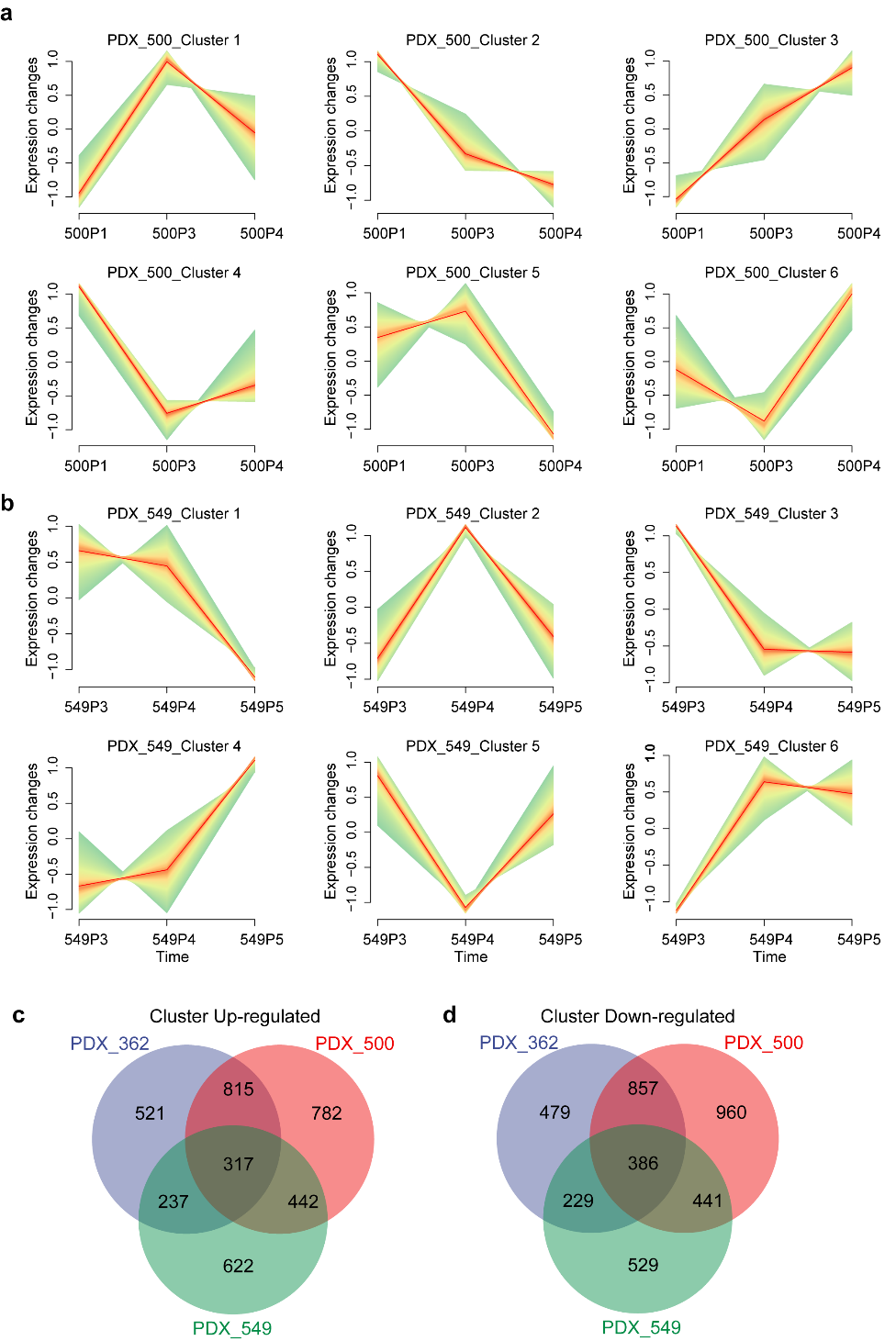


Figure. S9. Transcriptional dynamic analysis of acquired resistance to cetuximab related to Figure 6.

**a, b.** Clustering genes by similar expression profiles in PDX_500 (**a**) and PDX_549 (**b**) for time series samples. **c.** The Venn diagram shows the overlaps and differences between the genes in up-regulated trend clusters. **d.** The Venn diagram shows the overlaps and differences between the genes in down-regulated trend clusters.


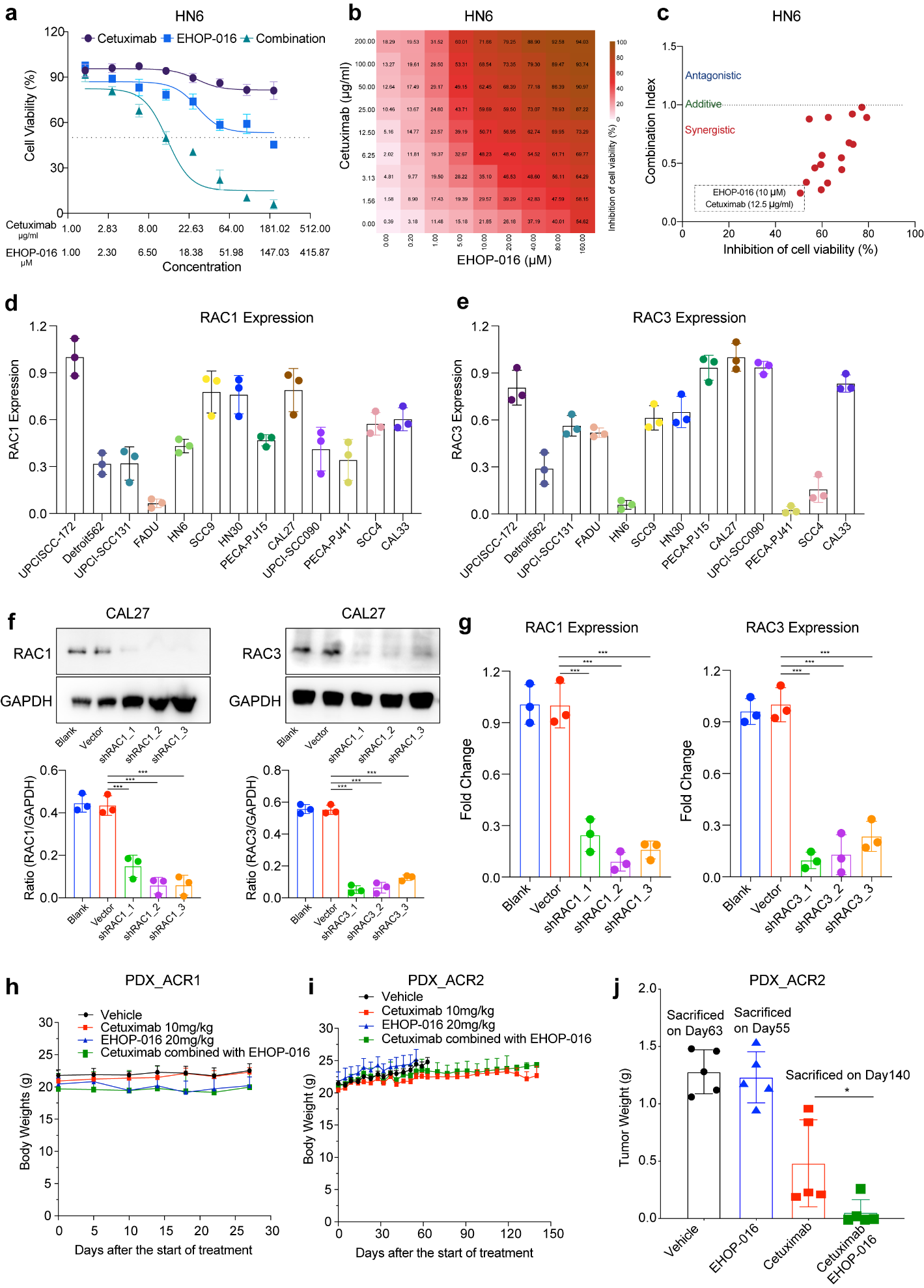


Figure. S10. Therapeutic intervention of RAC1 and RAC3 by EHOP-016 to overcome resistance to cetuximab in HNSCCs related to Figure 7.

**a.** Dose–response effects of cetuximab, EHOP-016 alone or in combination on the inhibition of cell viability in HN6 cells estimated after 3 days of drug treatment using CCK-8 assays. Each condition represents at least 3 biological replicates. **b.** The 9 × 9 heatmaps for the combination of cetuximab with EHOP-016 in HN6 cells. **c.** Combination index (CI) scores of EHOP-016 and cetuximab combination in HN6 cells*,* calculated at various effect levels (bottom). **d-e.** The graph depicts the expression of *RAC1* (**d**) and *RAC3* (**e**) in 13 HNSCC cell lines. **f-g.** Knockdown efficiencies of RAC1 or RAC3 in CAL27 cells were determined by Western blot (**f**) and qPCR (**g**). **h-i.** The body weight growth curve of each group in 2 cases of acquired resistance PDX models. **j.** Tumor weight of PDX_ACR2. Mean tumor volumes ±SD are plotted. **P*≤0.05; ***P*≤0.01; ****P*≤0.001.

Table S1.

Clinical data of patients from whom PDX models were established in the first PCT.

| Characteristic | No. patients | % |
| --- | --- | --- |
| Gender |  |  |
| Male | 33 | 67.35 |
| Female | 16 | 32.65 |
| Age(years) |  |  |
| Median(range) | 62 | (40-96) |
| Smoking history |  |  |
| Never smoker | 30 | 61.22 |
| Smoker | 19 | 38.78 |
| Primary site |  |  |
| Buccal mucosa | 7 | 14.29 |
| Tongue | 17 | 34.69 |
| Gingiva | 7 | 14.29 |
| Soft palate | 1 | 2.04 |
| Floor of mouth | 6 | 12.24 |
| Maxillary | 2 | 4.08 |
| Mandible | 4 | 8.16 |
| Oropharynx | 5 | 10.20 |
| Stage |  |  |
| Ⅰ | 1 | 2.04 |
| Ⅱ | 13 | 26.53 |
| Ⅲ | 23 | 46.94 |
| Ⅳ | 12 | 24.49 |
| Treatment status |  |  |
| Treatment-naïve | 36 | 73.47 |
| Post-treatment | 13 | 26.53 |

Table S2.

Percentage change of tumor volume in each case in the first PCT.

| ID | 21 days after first round treatment initiation | | Endpoint of the PCT | | Group |
| --- | --- | --- | --- | --- | --- |
|  | Response (%) | Efficacy assessment | Response (%) | Efficacy assessment |  |
| PDX_299 | 725.37 | mPD | 725.37 | mPD | intrinsic resistance |
| PDX_421 | 266.84 | mPD | 266.84 | mPD | intrinsic resistance |
| PDX_490 | 58.29 | mPD | 126.70 | mPD | intrinsic resistance |
| PDX_527 | 254.92 | mPD | 319.94 | mPD | intrinsic resistance |
| PDX_536 | 190.82 | mPD | 350.76 | mPD | intrinsic resistance |
| PDX_541 | 647.31 | mPD | 709.84 | mPD | intrinsic resistance |
| PDX_572 | 315.17 | mPD | 315.17 | mPD | intrinsic resistance |
| PDX_613 | 307.58 | mPD | 515.56 | mPD | intrinsic resistance |
| PDX_646 | 222.51 | mPD | 352.31 | mPD | intrinsic resistance |
| PDX_361 | 236.04 | mPD | 236.04 | mPD | intrinsic resistance |
| PDX_420 | 530.89 | mPD | 530.89 | mPD | intrinsic resistance |
| PDX_434 | 277.87 | mPD | 277.87 | mPD | intrinsic resistance |
| PDX_457 | 90.44 | mPD | 262.28 | mPD | intrinsic resistance |
| PDX_621 | 408.14 | mPD | 408.14 | mPD | intrinsic resistance |
| PDX_626 | 181.58 | mPD | 280.05 | mPD | intrinsic resistance |
| PDX_641 | 184.79 | mPD | 444.67 | mPD | intrinsic resistance |
| PDX_682 | 314.55 | mPD | 598.57 | mPD | intrinsic resistance |
| PDX_684 | 463.66 | mPD | 463.66 | mPD | intrinsic resistance |
| PDX_061 | 450.34 | mPD | 550.59 | mPD | intrinsic resistance |
| PDX_050 | 199.32 | mPD | 186.54 | mPD | intrinsic resistance |
| PDX_545 | 63.04 | mPD | 352.76 | mPD | intrinsic resistance |
| PDX_342 | -55.34 | mCR | -100.00 | mCR | sensitive |
| PDX_359 | -100.00 | mCR | -100.00 | mCR | sensitive |
| PDX_489 | -80.01 | mCR | -100.00 | mCR | sensitive |
| PDX_496 | -1.94 | mSD | -75.93 | mCR | sensitive |
| PDX_502 | -66.15 | mCR | -99.01 | mCR | sensitive |
| PDX_535 | -51.01 | mCR | -65.43 | mCR | sensitive |
| PDX_334 | -47.98 | mCR | -100.00 | mCR | sensitive |
| PDX_532 | -77.02 | mCR | -100.00 | mCR | sensitive |
| PDX_660 | 15.50 | mSD | -98.12 | mCR | sensitive |
| PDX_308 | -28.11 | mPR | 101.83 | mPD | acquired resistance |
| PDX_486 | -3.24 | mSD | 157.85 | mPD | acquired resistance |
| PDX_563 | -68.33 | mCR | 849.08 | mPD | acquired resistance |
| PDX_419 | -8.74 | mSD | 421.89 | mPD | acquired resistance |
| PDX_362 | -4.16 | mSD | 254.67 | mPD | acquired resistance |
| PDX_500 | -46.64 | mCR | 352.59 | mPD | acquired resistance |
| PDX_549 | -35.35 | mPR | 575.11 | mPD | acquired resistance |
| PDX_073 | -10.13 | mSD | 564.36 | mPD | acquired resistance |
| PDX_272 | -44.84 | mCR | 42.37 | mPD | individual differences |
| PDX_314 | 4.26 | mSD | 32.11 | mPD | individual differences |
| PDX_319 | 91.64 | mPD | 76.81 | mPD | individual differences |
| PDX_543 | -32.21 | mPR | 54.69 | mPD | individual differences |
| PDX_461 | -29.70 | mPR | -28.00 | mPR | DTP |
| PDX_666 | -47.69 | mCR | -54.45 | mCR | DTP |
| PDX_714 | -24.90 | mPR | -43.22 | mCR | DTP |
| PDX_701 | -100.00 | mCR | -100.00 | mCR | short observation time |
| PDX_084 | -38.86 | mPR | -38.86 | mPR | short observation time |
| PDX_014 | -61.56 | mCR | -53.76 | mCR | short observation time |
| PDX_124 | -39.88 | mPR | -48.42 | mCR | short observation time |

DTP: reversible drug-tolerant persister

mCR: complete response

mPR: partial response

mSD: stabilization

mPD: progressive disease

Table S3.

Percentage change of tumor volume in each case in the first PCT. Clinical data of patients from whom PDX models were established in the biomarker validation PCT.

| Characteristic | No. patients | % |
| --- | --- | --- |
| Gender |  |  |
| Male | 47 | 77.05 |
| Female | 14 | 22.95 |
| Age(years) |  |  |
| Median(range) | 62 | (24-88) |
| Smoking history |  |  |
| Never smoker | 46 | 75.41 |
| Smoker | 15 | 24.59 |
| Primary site |  |  |
| Buccal mucosa | 7 | 11.48 |
| Tongue | 20 | 32.79 |
| Gingiva | 13 | 21.31 |
| Soft palate | 5 | 8.20 |
| Floor of mouth | 4 | 6.56 |
| Maxillary | 1 | 1.64 |
| Mandible | 4 | 6.56 |
| Oropharynx | 7 | 11.48 |
| Stage |  |  |
| Ⅰ | 1 | 1.64 |
| Ⅱ | 9 | 14.75 |
| Ⅲ | 28 | 45.90 |
| Ⅳ | 23 | 37.70 |
| Treatment status |  |  |
| Treatment-naïve | 44 | 72.13 |
| Post-treatment | 17 | 27.87 |

Table S4.

Percentage change of tumor volume in each case in the biomarker validation PCT.

| ID | Response (%) | Efficacy assessment | Group |
| --- | --- | --- | --- |
| PDX_2479 | 680.32 | mPD | intrinsic resistance |
| PDX_2235 | 632.78 | mPD | intrinsic resistance |
| PDX_2086 | 571.73 | mPD | intrinsic resistance |
| PDX_2467 | 564.15 | mPD | intrinsic resistance |
| PDX_2110 | 509.44 | mPD | intrinsic resistance |
| PDX_2463 | 456.69 | mPD | intrinsic resistance |
| PDX_2345 | 453.12 | mPD | intrinsic resistance |
| PDX_2123 | 363.71 | mPD | intrinsic resistance |
| PDX_2103 | 359.75 | mPD | intrinsic resistance |
| PDX_2636 | 359.24 | mPD | intrinsic resistance |
| PDX_2543 | 327.47 | mPD | intrinsic resistance |
| PDX_2634 | 302.75 | mPD | intrinsic resistance |
| PDX_2121 | 288.91 | mPD | intrinsic resistance |
| PDX_2388 | 245.33 | mPD | intrinsic resistance |
| PDX_2324 | 229.88 | mPD | intrinsic resistance |
| PDX_2036 | 229.02 | mPD | intrinsic resistance |
| PDX_2154 | 206.75 | mPD | intrinsic resistance |
| PDX_2410 | 201.73 | mPD | intrinsic resistance |
| PDX_2195 | 163.89 | mPD | intrinsic resistance |
| PDX_2115 | 158.67 | mPD | intrinsic resistance |
| PDX_2148 | 150.63 | mPD | intrinsic resistance |
| PDX_2451 | 148.27 | mPD | intrinsic resistance |
| PDX_2119 | 130.01 | mPD | intrinsic resistance |
| PDX_2497 | 117.32 | mPD | intrinsic resistance |
| PDX_2055 | 111.78 | mPD | intrinsic resistance |
| PDX_2372 | 111.62 | mPD | intrinsic resistance |
| PDX_2548 | 101.78 | mPD | intrinsic resistance |
| PDX_2417 | 68.66 | mPD | intrinsic resistance |
| PDX_2375 | 29.23 | mSD | relatively stable |
| PDX_2231 | 15.77 | mSD | relatively stable |
| PDX_2309 | -1.49 | mSD | relatively stable |
| PDX_2319 | -3.85 | mSD | relatively stable |
| PDX_2426 | -4.59 | mSD | relatively stable |
| PDX_2553 | -6.09 | mSD | relatively stable |
| PDX_2111 | -7.99 | mSD | relatively stable |
| PDX_2439 | -8.30 | mSD | relatively stable |
| PDX_2084 | -11.31 | mSD | relatively stable |
| PDX_2391 | -13.66 | mSD | relatively stable |
| PDX_2232 | -18.27 | mSD | relatively stable |
| PDX_2642 | -18.37 | mSD | relatively stable |
| PDX_2090 | -18.92 | mSD | relatively stable |
| PDX_2105 | -21.03 | mPR | relatively sensitive |
| PDX_2178 | -22.25 | mPR | relatively sensitive |
| PDX_2104 | -24.66 | mPR | relatively sensitive |
| PDX_2603 | -26.91 | mPR | relatively sensitive |
| PDX_2491 | -28.65 | mPR | relatively sensitive |
| PDX_2352 | -28.89 | mPR | relatively sensitive |
| PDX_2452 | -29.12 | mPR | relatively sensitive |
| PDX_2539 | -32.67 | mPR | relatively sensitive |
| PDX_2540 | -35.17 | mPR | relatively sensitive |
| PDX_2556 | -36.47 | mPR | relatively sensitive |
| PDX_2205 | -37.20 | mPR | relatively sensitive |
| PDX_2459 | -38.72 | mPR | relatively sensitive |
| PDX_2532 | -38.82 | mPR | relatively sensitive |
| PDX_2332 | -39.21 | mPR | relatively sensitive |
| PDX_2283 | -47.47 | mCR | relatively sensitive |
| PDX_2120 | -76.67 | mCR | relatively sensitive |
| PDX_2419 | -97.09 | mCR | relatively sensitive |
| PDX_2445 | -97.44 | mCR | relatively sensitive |
| PDX_2601 | -100.00 | mCR | relatively sensitive |
| PDX_2122 | -100.00 | mCR | relatively sensitive |

mCR: complete response

mPR: partial response

mSD: stabilization

mPD: progressive disease

Table S5.

Primers used in this study.

| **Purpose** | **Gene** | | **Sequence (5'->3')** |
| --- | --- | --- | --- |
| Mutation validation | TP53 ^R150_R174^ | Forward | GACCTCTTAACCTGTGGCTTCTCC |
| Mutation validation | TP53 ^R150_R174^ | Reverse | CCCCAATTGCAGGTAAAACAGTCA |
| CNV validation | CDK1 | Forward | TGTCTCCTGATGGGCAAAGT |
| CNV validation | CDK1 | Reverse | ACATTGAGGCTCGAAGGGAA |
| CNV validation | ERBB2 | Forward | TGATGCGTGGTAGGGCATTT |
| CNV validation | ERBB2 | Reverse | CATGAGCAGCATTACCGTGC |
| CNV validation | ANKH | Forward | AGAACTAGACGAGGCTTGCG |
| CNV validation | ANKH | Reverse | TGGCACAAGACAAACCCGAT |
| CNV validation | HEPHL1 | Forward | ATTTCTCGAAAGAGGGCCCAA |
| CNV validation | HEPHL1 | Reverse | GTTCCATCCGTGAAGCGTCT |
| CNV validation | SLC6A3 | Forward | GTTTACACCTTTCCGTGCCG |
| CNV validation | SLC6A3 | Reverse | TCGCTGCACAGATCTACGTC |
| CNV validation | ANK3 | Forward | GAGCAACAAGATCCCTCCCC |
| CNV validation | ANK3 | Reverse | AATCGGAGCTGTTGTCCAGG |
| Gene expression | GAPDH | Forward | ATGACATCAAGAAGGTGGTG |
| Gene expression | GAPDH | Reverse | CATACCAGGAAATGAGCTTG |
| Gene expression | β-actin | Forward | GAGAAAATCTGGCACCACACC |
| Gene expression | β-actin | Reverse | GGATAGCACAGCCTGGATAGCAA |
| Gene expression | PBX1 | Forward | TTCCCATCTCAGCAACCCTT |
| Gene expression | PBX1 | Reverse | GGCTGACACATTGGTAGCAG |
| Gene expression | PODXL2 | Forward | CCTCTGCTACCTTGGGACAA |
| Gene expression | PODXL2 | Reverse | CAGATTGCTCCAGTCCTTGC |
| Gene expression | SIX2 | Forward | TTCCGCGAGCTCTACAAGAT |
| Gene expression | SIX2 | Reverse | CTTCTCCGCCTCGATGTAGT |
| Gene expression | GDF11 | Forward | CAACCCTGACCCATTCTCCT |
| Gene expression | GDF11 | Reverse | ACTCTGGTGCCCTGGTTATC |
| Gene expression | MAPK8IP1 | Forward | CTGTCAAGTACACGCTGGTG |
| Gene expression | MAPK8IP1 | Reverse | ACGGAGGCACAGTTGTCATA |
| Gene expression | SCARA3 | Forward | TGCCTTGTGCGTTACAGAAG |
| Gene expression | SCARA3 | Reverse | CAGGGCCAGGAAGAGGTAAA |
| Gene expression | CBX2 | Forward | TCCCAGATAGCTAGGCCAGA |
| Gene expression | CBX2 | Reverse | AGCAGTGAAGGTAGCACAGT |
| Gene expression | PARP3 | Forward | GCATCATGCCACATTCTGGT |
| Gene expression | PARP3 | Reverse | CCAGCTGACTTGCTGTTCTC |
| Gene expression | ATF3 | Forward | AGCCATTGGAGAGCTGTCTT |
| Gene expression | ATF3 | Reverse | AATGGCCAGTGTGTTAAGGC |
| Gene expression | KLF9 | Forward | TCAGTGTTCGAGGCTGTAGG |
| Gene expression | KLF9 | Reverse | AGGTGCGTCTAGAACTGAGG |
| Gene expression | MAPK15 | Forward | GAGGCCTTGGACCTCCTTAG |
| Gene expression | MAPK15 | Reverse | TACTCAGGCACAGAGAGCTG |
| Gene expression | PTPN18 | Forward | CTGATGGCCTGTCGAGAGAT |
| Gene expression | PTPN18 | Reverse | ATGTGACCTTGAGGGTCCTG |

Table S6.

shRNA sequences used in this study.

| **Gene** | **Name** | **Target Sequence (5'->3')** |
| --- | --- | --- |
| RAC1 | shRAC1_1 | CAGCTGGACAAGAAGATTATG |
| RAC1 | shRAC1_2 | GAGTCCTGCATCATTTGAAAA |
| RAC1 | shRAC1_3 | GTCCCTTGGAACCTTTGTACG |
| RAC3 | shRAC3_1 | GCTTGCTGATCAGCTACACGA |
| RAC3 | shRAC3_2 | GACGGGAAACCAGTCAACTTG |
| RAC3 | shRAC3_3 | GAGAATGTTCGTGCCAAGTGG |
